# Sustained Melanopsin Photoresponse Is Supported by Specific Roles of β-Arrestin 1 and 2 in Deactivation and Regeneration of Photopigment

**DOI:** 10.1016/j.celrep.2018.11.008

**Published:** 2018-11-27

**Authors:** Ludovic S. Mure, Megumi Hatori, Kiersten Ruda, Giorgia Benegiamo, James Demas, Satchidananda Panda

**Affiliations:** 1 Salk Institute for Biological Studies, 10010 North Torrey Pines Road, La Jolla, CA 92037, USA; 2 Keio University School of Medicine, Tokyo, Japan; 3 St. Olaf College, 1520 St. Olaf Avenue, Northfield, MN 55057, USA; 4 Lead Contact

## Abstract

Melanopsin-expressing intrinsically photosensitive retinal ganglion cells (ipRGCs) are indispensable for non-image-forming visual responses that sustain under prolonged illumination. For sustained signaling of ipRGCs, the melanopsin photopigment must continuously regenerate. The underlying mechanism is unknown. We discovered that a cluster of Ser/Thr sites within the C-terminal region of mammalian melanopsin is phosphorylated after a light pulse. This forms a binding site for β-arrestin 1 (βARR1) and β-arrestin 2. β-arrestin 2 primarily regulates the deactivation of melanopsin; accordingly, *βαrr2*^*–/–*^mice exhibit prolonged ipRGC responses after cessation of a light pulse. β-arrestin 1 primes melanopsin for regeneration. Therefore, *βαrr1*^*–/–*^ ipRGCs become desensitized after repeated or prolonged photostimulation. The lack of either β-arrestin atten-uates ipRGC response under prolonged illumination, suggesting that β-arrestin 2-mediated deactivation and β-arrestin 1-dependent regeneration of melanopsin function in sequence. In conclusion, we discovered a molecular mechanism by which β-arrestins regulate different aspects of melanopsin photoresponses and allow ipRGC-sustained responses under prolonged illumination.

## In Brief

The mechanism by which melanopsin-expressing retinal ganglion cells (mRGCs) tonically respond to continuous illumination is unknown. Mure et al. show that phosphorylation-dependent binding of β-arrestin 1 and 2 coordinately deactivate and regenerate melanopsin photopigment to enable sustained firing of mRGCs in response to prolonged illumination.

## Graphical Abstract

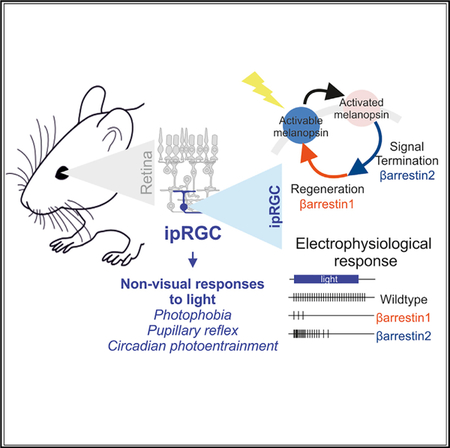

## INTRODUCTION

Melanopsin is an opsin class of photopigment that is expressed in a subset of retinal ganglion cells (RGCs), rendering them intrinsically photosensitive (ipRGCs). Melanopsin plays a pivotal role in non-image-forming (NIF) responses to light, including physiological adaptations (of pupil size, circadian rhythm, and activity) to ambient light (Nayak et al., 2007). In recent years, melanopsin has been shown to participate in a much broader range of processes, including developmental, visual, affective, and cognitive functions (Brown et al., 2010; LeGates et al., 2014; Rao et al., 2013). Many of the NIF visual responses mediated by melanopsin are sustained under continuous illumination, and such sustained responses are necessary for behavioral adaptation to ambient light. For example, the sustained response of melanopsin is necessary for light-induced activity suppression (negative masking) and photophobia in rodents. The lack of melanopsin, even in the presence of intact rod and cone function, attenuates masking under prolonged illumination (Johnson et al., 2010; Mrosovsky and Hattar, 2003). However, the mechanism or mechanisms underlying melanopsin function under continuous illumination are largely unknown.

Melanopsin photopigment uses *cis*-retinal as a chromophore. Upon light absorption, *cis*-retinal is photoisomerized to *all-trans*-retinal. Melanopsin is a G protein coupled receptor (GPCR) and photoactivated melanopsin signals by activating the Gα_q_/Gα_11_ class of G proteins (Isoldi et al., 2005; Panda et al., 2005; Qiu et al., 2005). It was previously shown that photoactivated mela-nopsin is also phosphorylated within its C-terminal region (Mure et al., 2016). Phosphorylation of GPCRs is known to trigger binding by arrestin proteins. Binding of arrestins to GPCRs hinders G protein activation, thereby terminating GPCR signaling (Gainetdinov et al., 2004). To enable subsequent photoresponses, the opsin-bound *all-trans*-retinal must be isomerized to or exchanged with *cis*-retinal. For rod and cone opsins, the retinoid cycle, in retinal pigment epithelial cells and Muller cells, is necessary to sustain a steady supply of *cis*-retinal and to allow image-forming vision under continuous illumination (Saari, 2012). Although there is evidence that melanopsin photores-ponses depend at least partially on regeneration of *cis*-retinal in the retinal pigment epithelium (Fu et al., 2005; Zhao et al., 2016), there is also evidence that melanopsin, like rhodopsin in *Drosophila,* can photoisomerize *all-trans-*retinal or exchange *all-trans*-retinal for *cis*-retinal in the presence of β-arrestin (Panda et al., 2005). Therefore, arrestins may participate both in terminating melanopsin signaling and allowing its regeneration under continuous illumination.

We recently discovered that a complex interaction between light-induced phosphorylation of melanopsin and the C-terminal region of melanopsin affects melanopsin response properties. Upon light activation, mouse melanopsin is phosphorylated in at least 9 Ser/Thr sites within its C-terminal cytoplasmic region (amino acid residues 380–397) (Blasic et al., 2014; Mure et al., 2016; Somasundaram et al., 2017), creating a potentially high-affinity binding site for arrestins. However, the long C-terminal region distal to this phosphorylation cluster delays deactivation. This delay imparts some inertia to the response, but it does not explain melanopsin function under prolonged illumination. Alternatively, mice expressing a mutant form of melanopsin that lacks these phosphorylation sites show prolonged activation after cessation of a light pulse, a phenotype that may be explained by reduced interaction with arrestins. Studies have suggested potential roles for arrestins in melanopsin responses (Cameron and Robinson, 2014; Panda et al., 2005); however, their specific function in ipRGC responses has not been investigated.

By using viral vectors to express melanopsin in random RGCs or in ipRGCs, we discovered that ipRGCs have an inherent ability to sustain light responses when exposed to repeated long pulses of light, whereas photoresponses of other RGCs reduce in magnitude (desensitize) or adapt to repeated photostimulation. β-arrestin 1 (bARR1) and β-arrestin 2 both interact with melanopsin, and their binding affinity increases upon light-dependent phos-phorylation of melanopsin. Mice lacking β-arrestin 2 or β-arrestin 1 show specific deficits in melanopsin deactivation and photopigment regeneration, respectively. As deactivation is a prerequisite for photopigment regeneration, in the absence of any one b-arrestin, ipRGCs fail to sustain photoresponses under prolonged illumination.

## RESULTS

### ipRGCs Have Unique Light Adaptation Properties that Are Independent of Melanopsin Structure

**Figure 1.**
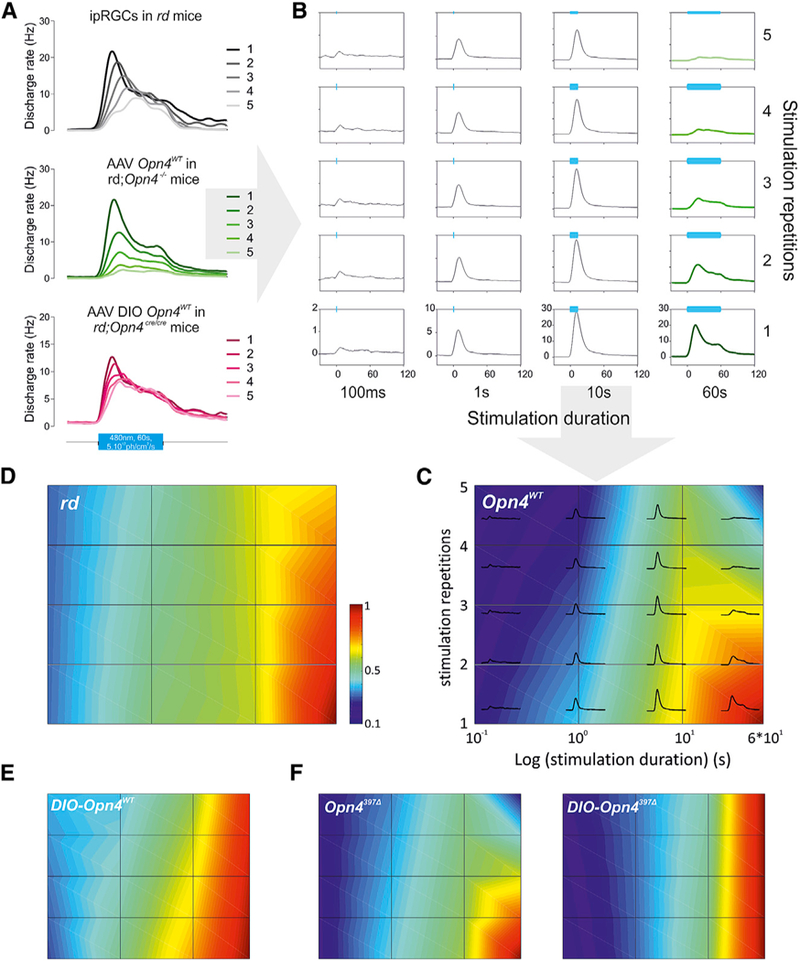
Melanopsin Photoresponses Are Affected by the Cellular Environment (A) When repetitively stimulated for 60 s (480 nm, 5 × 10^12^ photons/cm^2^/s), RGCs from *rd;Opn4*^*–/–*^mice transduced with Opn4^WT^ (middle, average responses, n = 58), but not ipRGCs from *rd* mice (top, n = 29) or ipRGCs from *rd;Opn4*^*cre/cre*^ mice conditionally transduced with Opn4^WT^ (bottom, n = 58), display reduced responses to successive light pulses. (B) Extracellular multielectrode array recordings of RGC responses to light (480 nm, 5 × 10^12^ photons/ cm^2^/s, 100 ms, 1 s, 10 s, and 60 s, 5 repetitions each) from the retina of adult *rd;Opn4*^*–/–*^ mice virally transduced with Opn4^WT^ that expresses the transgene indiscriminately in all RGCs (average responses n = 58). (C–E) LRAMs reporting duration of responses to the repetition protocol in *rd* (D), *rd;Opn4*^*–/–*^ transduced with Opn4^WT^ (C), and *rd;Opn4*^*cre/cre*^ mice conditionally transduced with Opn4^WT^ (DIO-Opn4) (E). (F) Differences in OPN4 photoresponse adaptation, whether expressed in RGCs or ipRGCs (DIO-Opn4), are also observed for *Opn4*^*397∆*^ mutant (n = 45 and n = 38, respectively).

To systematically examine melanopsin photoresponse, we monitored electrical responses of retina *ex vivo* using multielectrode arrays (MEAs) for extracellular recordings (Tu et al., 2005). We used retinas from retinal degeneration (*rd*) mice as they exhibit extensive degeneration of rod and cone photoreceptors in the outer retina. Thus, electrical responses of these retinas are predominantly from ipRGCs. In response to blue light (488 nm, 5 × 10^12^ photons/cm^2^/s), *rd* retinas produced a train of action potentials that were characteristic of melanopsin response, with slow onset (discharge rate rising above baseline by 2 SDs), sustained response, and slow deactivation (Figures 1A and S1A). Notably, at this irradiance level (5 × 10^12^ photons/cm^2^/s) used for all of the MEA experiments in the present study, the vast majority of ipRGCs is not affected by the recently described depolarization block (Emanuel et al., 2017; Milner and Do, 2017), and hence, most of them respond. To determine how this photoresponse adapts to different durations and repetitions of light stimuli, retinas were subject to increasing stimulus durations (100 ms, 1 s, 10 s, and 60 s), each repeated 5 times with a 3-min interpulse interval. The average light responses from the train of 20 stimuli were compiled in a light response adaptation map (LRAM). LRAM is a three-dimensional (3D) contour map of electrophysiological response duration to increasing duration of light stimuli plotted against the number of repetitions of the same stimuli (Figures 1B and 1C). Melanopsin photoresponses showed an increase in response magnitude with increasing light duration and a modest desensitization or reduc-tion in response to repeated stimulation, even with long-duration light stimulus (60 s). Such limited adaptation of melanopsin response is consistent with previous studies (Sexton and Van Gelder, 2015; Tu et al., 2005; Zhao et al., 2016).

To test whether the limited adaptation of melanopsin response is a property of ipRGCs or of melanopsin, we expressed melanopsin non-selectively in all RGCs of melanopsin-deficient mice (*rd;Opn4*^*–/–*^ ). We used a recombinant adeno-associated virus (rAAV) delivery system that has been optimized to express melanopsin primarily in the RGC layer of the mouse retina (Lin et al., 2008). This non-selective viral transduction approach expresses melanopsin in the vast majority of RGCs so that the extracellular responses recorded via the MEA largely reflect the responses of non-ipRGCs that now express melanopsin. Although heterologous expression of melanopsin imparted basic photosensitivity properties to RGCs, these cells showed unexpected desensitization properties (Figures 1A–1C). Similar to *rd* ipRGCs, cells from *rd;Opn4*^*–/–*^ retinas transduced with wild-type melanopsin (Opn4^WT^) increased their firing (duration and number of spikes) in response to stimuli of increasing duration. While these cells initially produced a relatively similar response to the repetition of identical stimuli (5 × 100 ms, 5 × 1 s), with longer-duration stimuli (10 s, 60 s) the responses dramatically diminished to the point at which the cells hardly responded to the fifth repetition of a 60-s stimulation. To rule out the possibility that the viral expression of melanopsin was artificially producing adaptation responses, we expressed melanopsin specifically in the native ipRGCs using conditional expression in *rd;Opn4*^*cre/cre*^ mice (a mouse strain with outer retina degeneration and where targeted integration of Cre leads to the loss of melanopsin expression [Hatori et al., 2008]). The retina of *rd;Opn4*^*cre/cre*^ mice injected with AAV DIO *Opn4*^*WT*^ (the double-floxed inverted open reading frame [ORF] must be recombined to be functional so that expression is achieved only in CRE-expressing cells) led to melanopsin expression in ipRGCs (Mure et al., 2016). When conditionally reintroduced in *rd;Opn4*^*cre/cre*^, melanopsin responses showed high resistance to desensitization and exhibited an overall response profile that was closer to the *rd* control (Figures 1A, 1C, and 1E). Overall, the initial responses to 100-ms or 1-s light pulses were equivalent in *rd* retina and in retina with virally expressed melanopsin, thus suggesting that melanopsin expression was not different between experiments (Mure et al., 2016). In summary, these differences in adaptation in non-ipRGCs and ipRGCs can be parsimoniously explained by the presence of interacting proteins in the ipRGCs that help sustain melanopsin response under repeated photostimulation.

The C-terminal region of melanopsin beyond the phosphorylation cluster (i.e., distal to amino acid [aa] 397) slows down or delays the deactivation of melanopsin after the termination of a light stimulus (Mure et al., 2016), either by steric hinderance or through interaction with an unknown factor. A version of melanopsin in which this C-terminal region has been deleted (OPN4^∆397^, which is truncated at aa 397, leaving mostly intact the phosphorylatable sites while removing the long C-terminal tail) exhibits accelerated deactivation and pigment regeneration, so that OPN4^∆397^ photoresponses to 60-s pulses of light increase linearly with light intensity (Mure et al., 2016). We hypothesized that if an interacting factor is necessary for the effect of the C terminus region and differential expression of this factor between ipRGCs and non-ipRGCs underlies the observed differences in adaptation, then LRAM of *Opn4*^∆*397*^ would be similar across RGC types. To test this hypothesis, we expressed *Opn4*^∆*397*^ mutant melanopsin using the AAV DIO expression cassette or non-selective AAV expression cassette to deliver *Opn4*^∆*397*^ to ipRGCs or to random RGCs in the retina of *rd;Opn4*^*cre/cre*^ mice. We systematically stimulated these transduced retinas with trains of light stimuli and constructed LRAMs for each condition. Differences in adaptation responses between specific and non-specific transduction of melanopsin were also observed with *Opn4*^*397∆*^ mutants (Figure 1F).

### Arrestins Modulate Melanopsin Response

Candidate partners of melanopsin to mediate response termination and resensitization are b-arrestin 1 and 2 (also called arrestin 2 and 3, respectively, and which, together with the visual arrestins [arrestins 1 and 4], make up the arrestin family of proteins). β-arrestin 1 and 2 are ubiquitous, multifunctional proteins that play pleiotropic roles in regulating GPCR responsiveness. They regulate the signal termination of GPCRs by uncoupling receptors from their cognate G proteins. They also participate in the sequestration of GPCRs by targeting receptors to clathrin-coated pits for endocytosis (Moore et al., 2007). GPCRs differ in their relative affinity for β-arrestin 1 and 2, and relative expression of these 2 β-arrestins has been suggested to modulate GPCR function (Oakley et al., 2000). We used a complementation assay (human embryonic kidney [HEK] cells; Figure S1B) to test physical interactions between arrestins and melanopsin upon light stimulation. A partial coding sequence of the β-galactosidase (β-gal) reporter was fused to either β-arrestin 1 or 2, and a complementary pep-tide (ProLink) was fused to the C terminus of mouse OPN4. In this assay, photoactivation results in the recruitment of the chimeric β-arrestin to the activated melanopsin, binding of the partial β-gal to the ProLink tag on OPN4, and reconstitution of a functional β-gal enzyme. Activity of the functional enzyme, which serves as a readout of the GPCR-arrestin interaction, is then quantified using a chemiluminescent substrate (Eglen, 2002).

**Figure 2.**
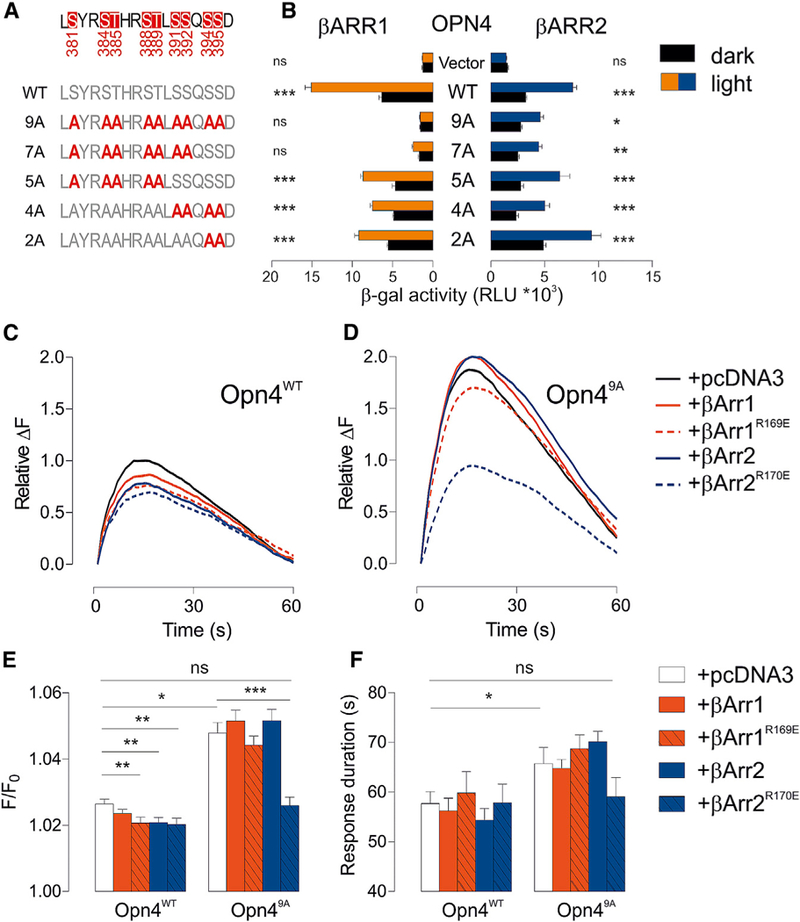
β-Arrestins Bind Melanopsin upon Light Activation and Participate in Its Deactivation (A) Mouse Opn4 mutants carrying alanine (A) at candidate Ser/Thr (S/T) phosphorylation sites. (B–F) In thisβ-galcomplementation assay (see Figure S1B), light-activated (5 min white light, 100 lx, red and blue bars) full-length Opn4 (WT) produces a robust chemiluminescent signal from OPN4-β-arrestin interaction compared to the dark control (black bars), while various phosphorylation-deficient Opn4 mutants show reduced barr1 or barr2 interaction (B) (2 way ANOVA, Bonferroni posthoc tests, *p < 0.05, ***p < 0.001, ns p > 0.05). Attenuation of melanopsin photoresponse in CHO cells transduced with either Opn4^WT^ or Opn4^9A^ and βArr1, βArr2, or their constitutively active mutant βa*rr1*^*R169E*^ and βa*rr2*^*R170E*^: average traces (C and D, respectively) and average peak amplitude (E) and time (F) (seconds ± SEMs) for the peak response to return to 5% of maximal response (n = 12–14, Student’s t test).

Compared to dark control, light stimulation of *Opn4*^*WT*^ significantly increased b-gal activity in the presence of either b-arrestin 1 or 2 (Figures 2A and 2B), thus demonstrating that melanopsin can interact with both β-arrestins in a light-dependent manner. To test whether phosphorylation of the melanopsin C terminus is required for β-arrestin recruitment, we expressed mutant versions of OPN4 in which S/T residues within the aa 381–397 region were replaced with non-phosphorylatable alanine (*Opn4*^*2A*^, *Opn4*^*4A*^, *Opn4*^*5A*^, *Opn4*^*7A*^, and *Opn4*^*9A*^) (Figure 2A) (Mure et al., 2016). The increasing loss of phosphorylation sites blunted light-dependent interactions between melanopsin and arrestins. *Opn4*^*7A*^ and *Opn4*^*9A*^ mutants showed reduced light-dependent interaction with β-arrestin 2 and no light-dependent interaction with β-arrestin 1.

To determine whether phosphorylation of the cytoplasmic region of mouse OPN4 is functionally important for the interaction with arrestin in melanopsin photoresponse, we expressed *Opn4*^*WT*^ or *Opn4*^*9A*^ in Chinese hamster ovary (CHO) cells (Figures 2C and 2D) in combination with β-arrestin 1, β-arrestin 2, or constitutively active versions of these arrestin proteins (phos-phorylation-independent b-arrestins, *βArr1*^*R169E*^ and *βArr2*^*R170E*^) (Celver et al., 2002; Kovoor et al., 1999). We then monitored light-dependent (488 nm, 500 mW excitation laser) increases in cytosolic Ca^2+^ in a fluorescent imaging plate reader (FLIPR) assay (Pulivarthy et al., 2007). In cells expressing Opn4^WT^, transient increases in Ca^2+^ fluorescence returned to 5% of the peak value in 57.7 ± 2.4 s. Response magnitude and duration were increased in cells expressing the phosphorylation-defective version of Opn4, with the *Opn4*^*9A*^ peak lasting for 65.7 ± 3.3 s (Figures 2E and 2F). Overexpression of β-arrestins with Opn4^WT^ caused a modest but significant decrease in response amplitude. When overexpressed with *Opn4*^*9A*^, however, only the constitutively active β-arrestin 2 (*βArr2*^*R170E*^) reduced the response peak. The phosphorylation-independent arrestin restored the responses of *Opn4*^*9A*^ to levels similar to those observed with Opn4^WT^, demonstrating the requirement of phosphorylation for normal β-arrestin interaction. However, the light-induced and melanopsin-mediated Ca^2+^ response in CHO cells could not be assessed under repeated photo-stimulation because restoration of intra-cellular calcium stores becomes limiting in this system.

### Loss of Arrestin Alters Melanopsin Adaptation/Resensitization

**Figure 3.**
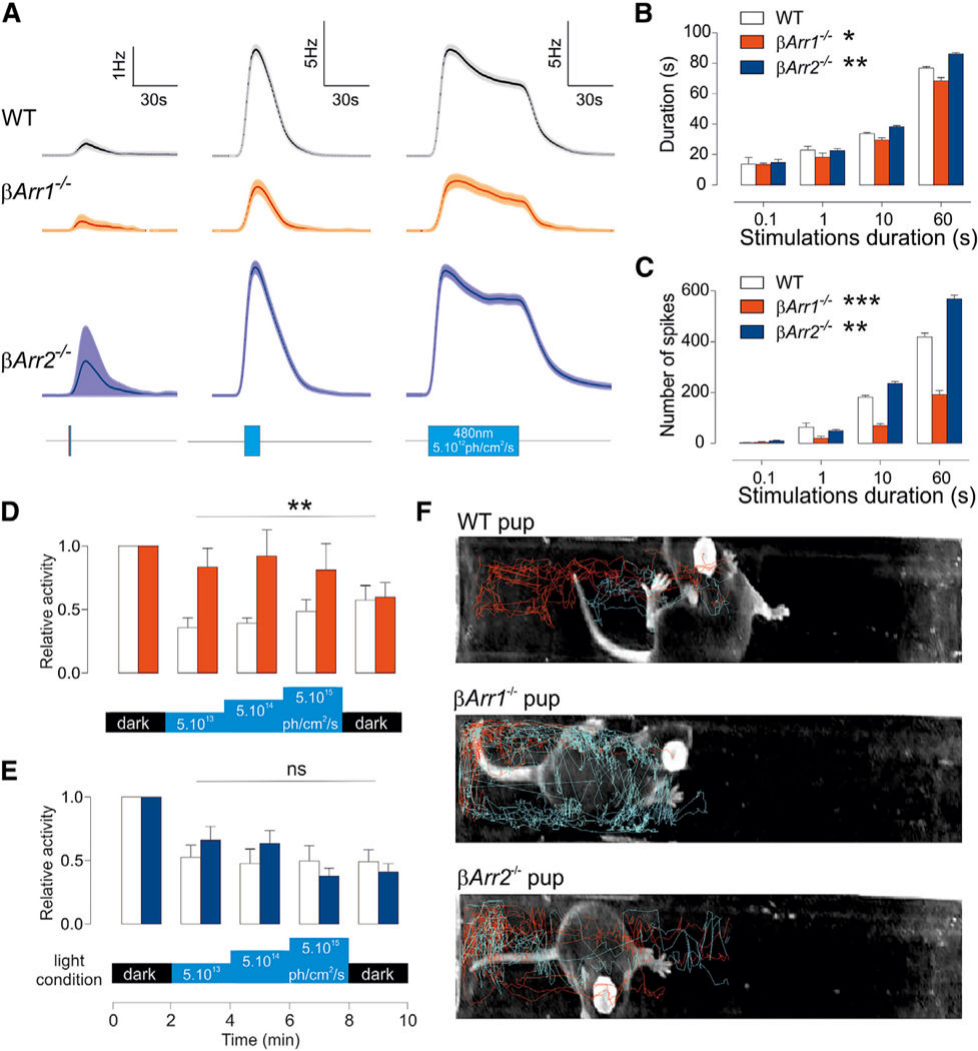
β-Arrestins Deficiency Affects Different Aspects of ipRGC Photoresponses and Translates at the Behavioral Level in the Pup (A–E) Light responses for WT (n = 284), b*arr1*^*–/–*^(n = 83), and *βarr2*^*–/–*^(n = 458) pup retinas. Average traces (A, 1 min, 480 nm, 5 × 10^12^ photons/cm^2^/s), response duration (B) (ANOVA, p = 0.03 and 0.005 for *βarr1*^*–/–*^and *βarr2*^*–/–*^, respectively), and number of spikes fired (C) (ANOVA, p = 6 × 10 ^5^ and 0.004 for *βarr1*^*–/–*^ and *βarr2*^*–/–*^, respectively) are shown (3–5 pups of each genotype). Negative phototaxis assay; average movement measured in response to different light conditions (2 min each, successive sequence: dark, 480 nm light at 5 × 10^13^, 5 × 10^14^, and 5 × 10^15^, and dark) in *βarr11*^*–/–*^ (n = 6, D) and *βarr21*^*–/–*^ (n = 7, E) compared to their WT littermates (n = 5 and 7, respectively, ANOVA, p = 0.0021 and 0.73 for b*arr11*^*–/–*^ and β*arr21*^*–/–*^, respectively, compared to their respective controls). (F) Representative movement of WT, *βarr11*^*–/–*^, and *βarr21*^*–/–*^ in the test tubes in darkness (first 2 min, red) and during the following illumination (last 6 min, blue).

To understand the role of β-arrestins in native ipRGC photoresponse, we carried out MEA extracellular recordings of light-induced electrical activity using ipRGCs from the retinas of ba*rr1*^*–/–*^ or ba*rr2*^*–/–*^ animals. Mice lacking both b-arrestins are non-viable, but single mutants have modest defects in the function of several GPCRs (Bohn et al., 1999; Conner et al., 1997), suggesting that βa*rr1* and βa*rr2* are functionally redundant, at least in part. Since neonatal mice have fully functional ipRGCs before the establishment of a functional rod and cone photoreceptor system (Sekaran et al., 2005), the photoresponses of retinas from postnatal days 6–9 (P6–P9) of βa*rr1*^*–/–*^or ba*rr2*^*–/–*^pups were evaluated. Extracellular recording of photoresponses (480 nm, 5 3 10^12^ photons/cm^2^/s) in the retinas from WT pups showed a characteristic train of action potentials, with slow onset (discharge rate rising above baseline by 2 SDs), sustained response, and slow deactivation (Figures 3A and S2A). Progres-sive increase of the duration of the stimulations from 100 ms to 1 s, 10 s, and 60 s allowed us to systematically measure the ipRGC activation threshold, response latency, duration, and average level. The latency to respond to a subsaturating stimulation is determined by how fast the cell potential reaches the firing threshold. This is a function of the pool of active melanopsin, which itself depends on melanopsin activation and deactivation rates and regeneration. While brief light pulses (100 ms or 1 s) probe the basic photoresponse properties of melanopsin, long stimulation (R10 s) evaluates regeneration capacities, as the tonic melanopsin response is established at a level dependent on the pool of available photopigment. Responses from b*arr1*^*–/–*^ or b*arr2*^*–/–*^ retinas were not significantly different from those of WT retinas upon stimulation with 100-ms or 1-s light pulses (Figures 3B and 3C), suggesting that the melanopsin photopigment and its single photon responses are largely unaffected by the absence of a single b-arrestin. However, in response to 10 or 60 s of light, the contribution of individual β-arrestins to the ipRGC photoresponse was differentiated. Compared to WT retinas, the latency to response was significantly longer in β*arr1*^*–/–*^retinas, while it was faster in b*arr2*^*–/–*^retinas (2.0 ± 0.34 s in WT, 2.4 ± 0.37 s in b*arr1*^*–/–*^, and 1.67 ± 0.16 s in b*arr2*^*–/–*^for 60-s stimulations; Figure S2C). The average response under prolonged illumination (10 s, 60 s) was reduced in b*arr1*^*–/–*^retinas, whereas it was increased in b*arr2*^*–/–*^retinas (Figures 3A–3C and S2A). ipRGCs of b*arr1*^*–/–*^ mice displayed significantly slower (Figure S2C), smaller (Figure 3C), and shorter photoresponses (Figure 3B) upon 10-s or 60-s stimulations. Such response attenuation in b*arr1*^*–/–*^ retinas under prolonged illumination suggest a reduced pool of photoactivable pigment, which would arise from impaired regeneration. However, b*arr2*^*–/–*^ retinas displayed response properties that were the opposite of those observed in b*arr1*^*–/–*^ ipRGCs: shorter latency to respond, higher magnitude of response (or number of spikes), and prolonged response even after cessation of the light pulse (Figures 3A–3C, S2A, and S2C).

Neonatal ipRGCs (P8) can be separated into 3 types (I–III), based on their electro-physiological responses (Tu et al., 2005). We tested whether the differences observed in *βarr1*^*–/–*^ or *βarr2*^*–/–*^ mice could be due to an alteration in subtype distribution. Response sensitivity can be used to discriminate between type I/III and type II cells, whereas the activation latency separates type I and type III cells (Sexton and Van Gelder, 2015; Tu et al., 2005). In the present study, the irradiance used (5 × 10^12^ photons/cm ^2^/s ^1^) did not allow us to distinguish types I and III based on latency, but we observed a cluster of cells with very delayed responses that resemble type II. The proportion of ‘‘short’’ and ‘‘delayed’’ latency cells was not affected by the absence of either β-arrestin (Figure S2D). The distribution of stimulation durations at which the ipRGCs start to respond, an indication of sensitivity, was not altered either (Figure S2E). In summary, these results indicate that the change in ipRGC light sensitivity properties in *βarr1*^*–/–*^ or *barr2*^*–/–*^ mice could not be explained by developmental alterations in ipRGC subtype distribution. Rather, these alterations reflect the roles of specific β-arrestins in melanopsin function.

### Neonatal Behavioral Response to Ambient Light Is Shaped by β-Arrestin

We next tested whether the contribution of individual arrestins to melanopsin photoresponse is relevant to the animal’s adaptation to the light environment. A behavioral manifestation of melanopsin photoreception in young rodent pups is negative phototaxis or photophobia (Johnson et al., 2010). As mentioned earlier, neonatal mice (<P14) have fully functional ipRGCs before the establishment of a functional rod and cone photo-receptor system (Sekaran et al., 2005). By this age, ipRGC axons have already innervated major brain targets implicated in photophobia (Jones et al., 2013). These pups show exploratory activity under darkness. A light stimulus (2 min steps at 5 × 10^13^, 5 × 10^14^, and 5 × 10^15^ photons/cm^2^/s) triggers an avoidance response, in which the pup turns its head, moves away from the light source, and stops activity as long as the light stimulus continues. WT pups showed a strong aversion to blue light ( 50% reduction in activity) that matches the peak spectral sensitivity of melanopsin (Figures 3D–3F). *βarr1*^*–/–*^ pups were not averse to light (Figure 3D), a phenotype that was also observed in *Opn4*^*–/–*^pups or WT pups treated with melanopsin antagonist (Johnson et al., 2010; Jones et al., 2013). *βarr1*^*–/–*^pups continued to be active during the light stimuli. This reduced behavioral aversion to light in *βarr1*^*–/–*^pups is consistent with the reduced photoresponses measured from their retinas via MEA. In contrast, *βarr2*^*–/–*^pups, like the WT pups, reduced their activity immediately after light ON (Figures 3E and 3F).

### Arrestins Shape Melanopsin Photoresponses in Adult Mice

**Figure 4.**
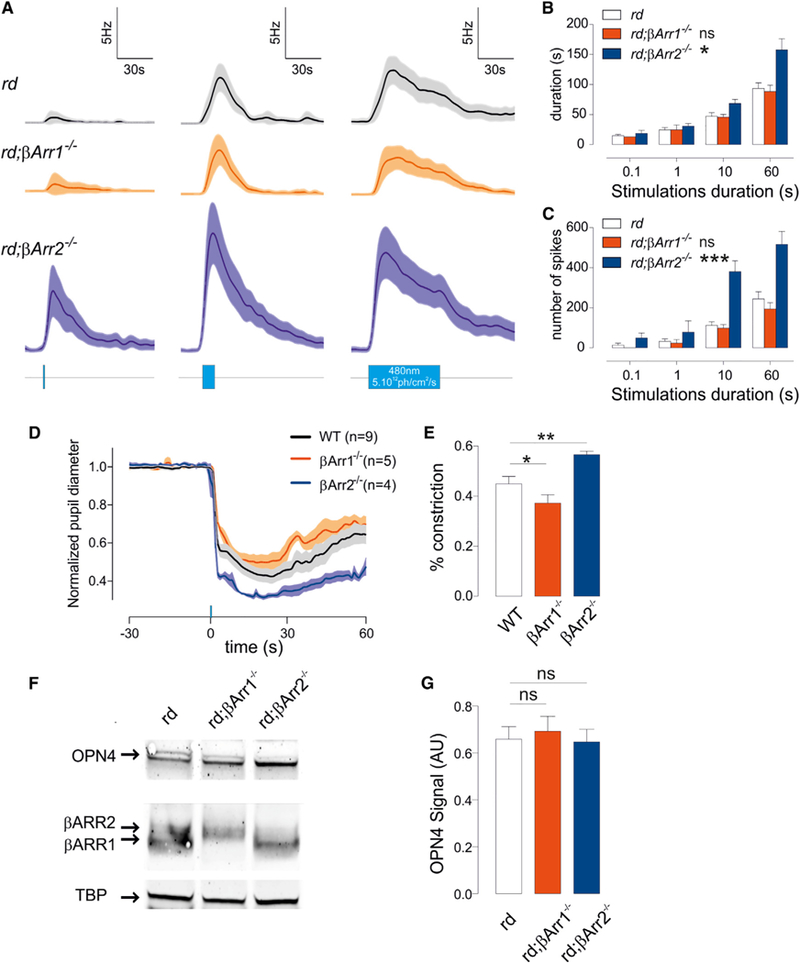
β-Arrestins Deficiency Affects Different Aspects of ipRGC Photoresponses and Translates at the Behavioral Level in the Adult (A–D) In adults *rd, rd;βarr1*^*–/–*^ oand *rd;βarr2*^*–/–*^ mice ipRGC responses to light (*rd* n = 32, *rd;*β*arr1*^*–/–*^ n = 32, and *rd;βarr2*^*–/–*^ n = 52) from 2–3 mice, average traces (A, 1 s, 10 s, and 1 min, 480 nm, 5 3 10^12^ photons/cm^2^/s), (B) response duration (ANOVA, *rd; βarr2*^*–/–*^ : *p = 0.03); and number of spikes fired (ANOVA, *rd; βarr2*^*–/–*^ : ***p = 0.0002) (C) are mirrored by pupillary constriction (D). PLR average traces response to 1 s stimulation for *WT,* n = 9; *βarr1*^*–/–*^, n = 5; *βarr2*^*–/–*^, n = 4. (E–G) Average constriction measured during the 1 min after a 1-s stimulation (E). Melanopsin protein level is not altered in *rd;βarr1*^*–/–*^ or *rd;βarr2*^*–/–*^ retinas (blot, F, and average protein level, G; n = 3).

To test the contribution of b-arrestins to melanopsin photoresponse in adult mice, we bred the *βarr* knockout mice with the *rd* mice to generate *rd;βarr1*^*–/–*^ and *rd;βarr2*^*–/–*^ mice. These mice, like the *rd* mice, showed rod and cone outer retina degeneration by 4–6 weeks of age. Thus, light responses from adult retina mostly reflect responses from ipRGCs that are deficient in individual β-arrestins. As seen for the retinas of *βarr2*^*–/–*^ pups (Figures 3A–3C and S2A–S2C), the response properties of *rd;βarr2*^*–/–*^ retina differed from control (Figures 4A–4C and S3A). When *rd;βarr2*^*–/–*^ mice were exposed to long-duration light pulses, their responses were characterized by short latency and high magnitude. In response to brief pulses of light, the duration of response after light OFF was significantly prolonged in *rd;βarr2*^*–/–*^ mice. The *rd;βarr1*^*–/–*^ retina tends to show a reduced sensitivity to light with a reduced amplitude of response. Overall, the ipRGC firing properties of the adult retina paralleled those of the neonatal retina, but there were differences in the magnitude of the effect of β-arrestin deficiency. This may partly be explained by the gradual change in the composition of ipRGC sub-types and in the firing properties of ipRGCs during retina maturation in young adults (Tu et al., 2005).

Similar to neonatal light response analyses, we verified that the changes in ipRGC light sensitivity properties in *rd;βarr1*^*–/–*^ or *rd;βarr2*^*–/–*^ mice could not be explained by the alteration of 1 particular ipRGC subtype. We performed k-means clustering analysis on ipRGC responses to 1-min stimuli. Sorted into 2 clusters, cells from each genotype revealed response profiles and distributions (Figure S3B) similar to types II and III ipRGCs (in *rd*: n = 22 and 16, in *rd;βarr1*^*–/–*^ : n = 22 and 10, and in *rd;βarr2*^*–/–*^ : n = 37 and 12 for type II and type III, respectively), as reported by Tu et al. (2005). Both subtypes displayed the same longer responses in *rd;βarr2* ^*–/–*^ mice compared to the equivalent groups in *rd* mice (Figure S3C). These results indicated that alterations observed in *rd;βarr1*^*–/–*^ or *rd;βarr2*^*–/–*^ mice reflect the roles of specific β-arrestins in melanopsin function.

As seen recently (Sexton and Van Gelder, 2015), the loss of function of some melanopsin-interacting proteins has very little effect on the behavioral response of adult mice to light. A reliable behavioral readout of electrical response properties of melanopsin function is the pupillary light reflex (PLR). In normal mice, rods, cones, and melanopsin mediate PLR (Hayter and Brown, 2018; Keenan et al., 2016; Lucas et al., 2003), while in *rd* mice, all PLR response is mediated by melanopsin (Hattar et al., 2003; Panda et al., 2003). We measured PLR in mice defi-cient for β-arrestin. In response to a 1-s pulse of light (480 nm, 1 × 10^14^ photons/cm^2^/s), the magnitude of pupil constriction (and subsequent pupil relaxation after light OFF) paralleled the magnitude of electrical response and duration of light response in the respective genotypes of mice (Figures 4D, 4E, and S4A). Under these conditions, melanopsin largely mediates PLR, as WT and *rd* mice responses were not significantly different (Figures S4C and S4D). The maximal constriction in WT retina was 58.8% ± 0.04%, relaxing to 35.3% ± 0.05% within 1 min of cessation of the light pulse. *βarr1*^*–/–*^ pupils showed attenuated constriction (51.1% ± 0.04%) and relaxed faster than WT (30.0% ± 0.04% after a 1-min pulse); a similar effect was observed in *rd;βarr1*^*–/–*^ compared to *rd* mice (Figures S4E and S4F). In contrast, *βarr2*^*–/–*^ mice showed more pronounced constriction and a severe deficit in relaxation (69.1% ± 0.01% and 54.6% ± 0.03%, respectively).

To eliminate the possibility that the altered responses we observed in β-arrestin 1- and β-arrestin 2-deficient animals could reflect a different level of melanopsin protein, we quantified melanopsin protein levels in *rd*, *rd;βarr1*^*–/–*^, and *rd;βarr2*^*–/–*^ retinas. Mice lacking either b-arrestin 1 or β-arrestin 2 displayed very similar levels of melanopsin protein to their intact *rd* controls (Figures 4F, 4G, and S4G). In addition, we verified that at the level of the entire retina, lack of 1 β-arrestin isoform did not affect the levels of the remaining isoform (Figure S4H).

### Arrestin in Adaptation of Melanopsin Response

**Figure 5.**
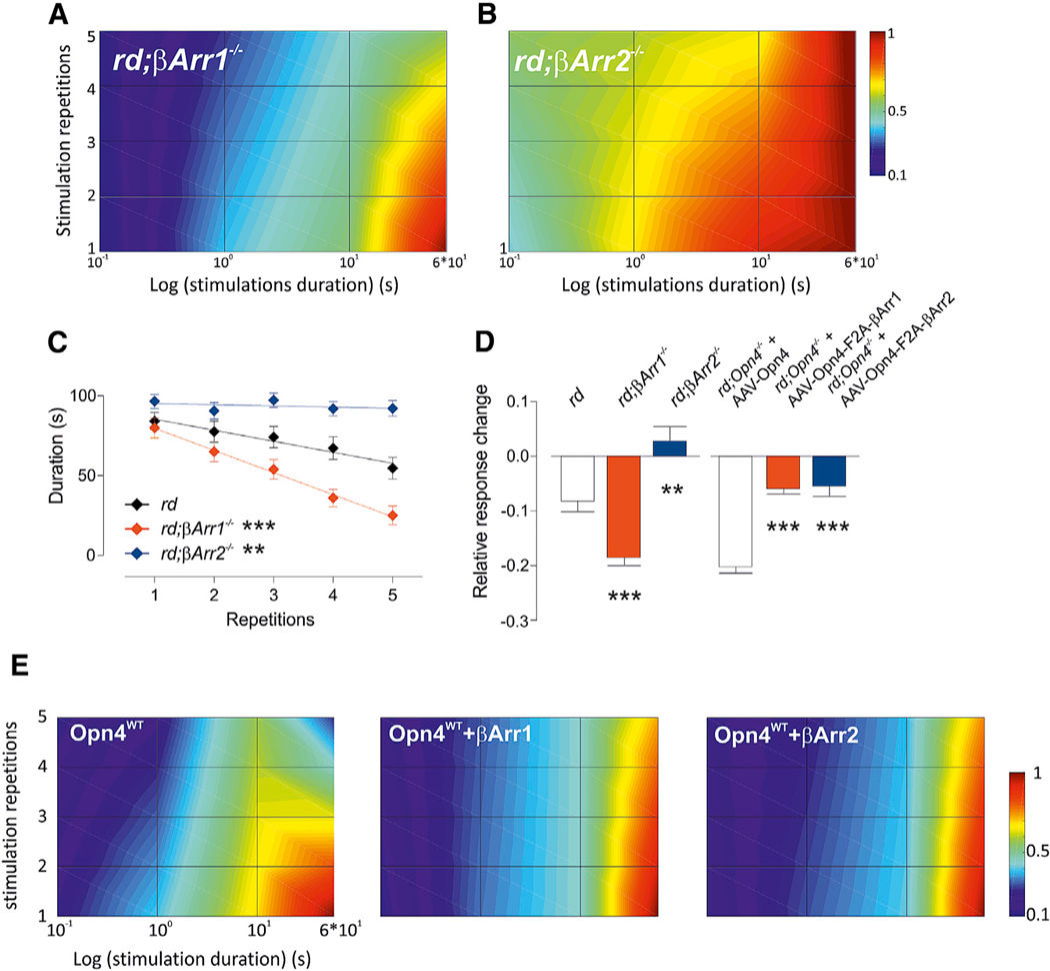
βArr2 Is Required for Signal Termination, while bArr1 Participates in Receptor Recycling (A and B) ipRGCs responses profiles to light (MEA, nm, 5 × 10^12^ photons/cm^2^/s, 100 ms, 1 s, 10 s, and 60 s, 5 repetitions each) in retinas from *rd;βarr1*^*–/–*^ (A) and *rd;βarr2*^*–/–*^ (B) mice. (C) *rd;βarr1*^*–/–*^ ipRGCs display strong adaptation when repetitively stimulated for 1 min (slopes different from 0: rd, **p = 0.005, rd;βArr1, ***p = 0.0002, rd;βArr2, p = 0.52, ns; slopes different from rd: rd;bArr1, ***p = 0.0007, rd;βArr2, **p = 0.004). (D) Adaptation rate (relative response change from one stimulation to the other, average ± SEM) observed in the β-arrestin-deficient retina model (*rd* compared to *rd;βArr1* and *rd;βArr2*) and β-arrestin overexpression model (AAV Opn4^WT^ compared to AAV Opn4^WT^-F2A-bArr1 or 2) in response to 5 repetitions of an identical 1-min-long light stimulus (MEA, 480 nm, 5 × 10^12^ photons/cm^2^/s) (t test, A2 versus A2-F2A-βarr2,***p = 1.9 3 10 ^5^; A2 versus A2-F2A-βarr1, ***p = 5.× 3 10 ^10^; *rd* versus *rd;βarr1*^*–/–*^, ***p = 5.5 × 10 ^5^; *rd* versus *rd;βarr2*^*–/–*^, **p = 0.003). (E) LRAM from *rd*;*Opn4*^*–/–*^ mice retinas transduced with Opn4^WT^ (n = 57), Opn4^WT^*-F2A-βArr1* (n = 45), and Opn4^WT^*-F2A-βArr2* (n = 111) (100 ms, 1s, 10 s, and 1 min, 480 nm, 5 × 10^12^ photons/ cm^2^/s).

While the results presented so far describe the effects of arrestins on acute responses to light, to test their roles in adaptation to repeated light stimulation, we subjected retinas of *rd;βarr1*^*–/–*^ or *rd;βarr2*^*–/–*^ mice to trains of light pulses (as described in Figure 1) and generated LRAM plots from the data. The reduced responses observed in *βarr1*-deficient animals, while their melanopsin level is similar to WT mice, suggests that β-arrestin 1 may contribute to pigment regeneration. Thus, we hypothesized that light responses from the *rd;βarr1*^*–/–*^ retina should adapt faster to repetitive light stimulation, which is comparable to the adaptation seen when melanopsin is expressed in random RGCs. As expected, when subjected to successive identical 60-s stimulations, *rd;βarr2*^*–/–*^ oretinas showed prolonged responses that resisted adaptation, irrespective of the number of preceding stimulations (Figures 5B–5D and S5B). Retinas from *rd;βarr1*^*–/–*^ omice, however, showed a normal response to the first 60-s pulse of light, as in *rd* controls, but responses gradually diminished as 4 more pulses of light were applied (Figures 5A, 5C, and 5D). This trend in photoresponses was also observed in PLR (Figures S4A and S4B).

**Figure 6.**
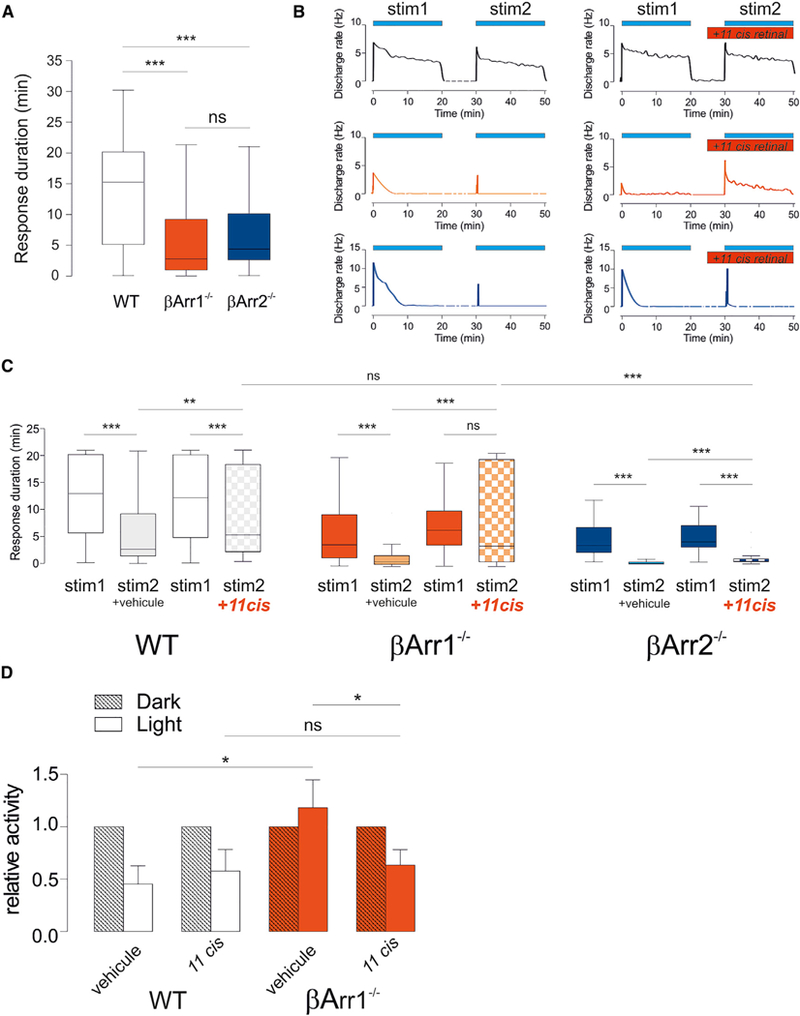
β-Arrestins Are Required for Melanopsin Continuous Signaling (A) Duration of responses to continuous 20-min illumination (MEA, 480 nm, 5 × 10^12^ photons/cm^2^/s) (median ± 25th/75th percentiles, n = 67, 300, and 112 for WT, *βarr1*^*–/–*^ and *βarr2*^*–/–*^ respectively; 3–4 pups each, Mann-Whitney test, *βarr1*^*–/–*^ and *βarr2*^*–/–*^ both ***p < 0.0001). (B and C) Example of individual responses from *WT, βarr1*^*–/–*^ and *βarr2*^*–/–*^ to 2 successive 20-min stimulations (stim1 and stim2, 480 nm, 5 × 10^12^ photons/cm^2^/s) with or without addition of 11-*cis*-retinal in the medium (B) and corresponding median duration of the responses (C) (WT n = 64 and 128, *βarr1*^*–/–*^ n = 117 and 95, *βarr2*^*–/–*^ n = 46 and 65, for stim1 and stim2, respectively; 2 pups each, **p < 0.01 and ***p < 0.001, Wilcoxon signed rank test between stim1 and stim2; Mann-Whitney test between experiments or genotypes). (D) Negative phototaxis assay; average movement measured in response to different light conditions (2 min of darkness followed by 2 min of 480-nm light at 5 × 10^14^ photons/cm^2^/s) in WT and *βarr1*^*–/–*^ pups. The pups are administered either *11-cis*-retinal (WT n = 6 and *βarr1*^*–/–*^ n = 5) or its vehicle only 5–10 min before the assay (WT n = 4 and *βarr1*^*–/–*^ n = 4) (*p < 0.05, one-tailed Student’s t test).

These results suggest that β-arrestin 2 may support melanopsin deactivation, but we cannot rule out the possibility that β-arrestin 2 is also involved in pigment regeneration. In the absence of normal deactivation, photoactivated melanopsin, even if present in progressively smaller quantities with each light pulse, would be sufficient for sustained activation of the downstream signaling cascade, as signal amplification steps would mask potential deficits in regeneration. To address this issue, we probed the retina with long light pulses, so that regeneration would be limiting. When submitted to long, continuous illumination (20 min, 480 nm, 5 × 10^12^ photons/cm^2^/s), the retinas of WT and *βarr1* ^*/*^ pups showed results that were consistent with the repetitive pulse protocol (Figures 6A and S5B). The initial transient peak response in *βarr1*^*–/–*^ was attenuated >50% relative to WT. While a large number of cells from WT retinas were able to respond during most of the stimulation (median response 915 s), the duration of responses from *βarr1*^*–/–*^ retinas were substantially reduced (median 168 s). In other words, WT retinas resisted desensitization, whereas *βarr1*^*–/–*^ retinas were rapidly desensitized. As seen with the 60-s light pulse (Figures 3A, 4A, and 5B), *βarr2*^*–/–*^ retinas initially mounted a robust response. However, responses under continuous illumination did not persist (median 263.5 s) (Figure 6A). Taking into account the responses to both short and long light pulses, it appeared that under ‘‘short’’ stimulations (±60 s), deficits in deactivation resulted in a temporarily increased response, which masked the reduced regeneration efficiency. Under repeated pulses of 60 s or prolonged illumination of several minutes, regeneration was limiting, and hence response durations were reduced in *βarr2*^*–/–*^ mice.

To test whether co-expression of arrestin with melanopsin in random RGCs can modify their adaptation (or regeneration) properties or rates, we used rAAV2/2 vectors to express either Opn4^WT^ alone or Opn4^WT^ with β-arrestin 1 or β-arrestin 2 (linked by a self-cleaving 16-amino acid 2A peptide of foot and mouth disease virus, F2A) in the retinas of adult *rd;Opn4*^*–/–*^ mice. The F2A linkage system supports nearly equal expression of both proteins (Szymczak et al., 2004). As expected, RGCs transduced with *Opn4*^*WT*^*-F2A-βarr2* displayed shorter responses, which is consistent with a faster signal termination (Figures S6C and S6D). *Opn4*^*WT*^*-F2A-*β*arr1* RGCs displayed responses that were similar to *Opn4*^*WT*^ alone (Figures S6A–S6D), but repetition of the same stimulus revealed a decreased adaptation rate when βa*RR1* was overexpressed (Figures 5D, S6E, and S6F). Retinas with *Opn4*^*WT*^*-F2A-βarr2* also showed sustained responses when repetitively subjected to 60-s light pulses.

To further assess the role of b-arrestin in ipRGCs resensitization, we recorded responses to long light stimulation (20 min) while adding 11-*cis*-retinal (the active form of the melanopsin chromophore) to the medium (Figures 6B and 6C). We hypothesized that if β-arrestin 1 plays a critical role in melanopsin regeneration (*all-trans* isomerization to or exchange with *11-cis-*retinal), then providing exogenous 11-*cis*-retinal should rescue light response in *βarr1*^*–/–*^ animals. After a first 20-min light stimulation, most ipRGCs from WT retinas were able to respond to a subsequent 20-min stimulation. In contrast, responses from *βarr1*^*–/–*^ and *βarr2*^*–/–*^ retinas to a second stimulation were further reduced (Figures 6B and 6C). When *11-cis*-retinal was added to the medium between the 2 stimulations, the duration of the responses to the second stimulation increased in the 3 genotypes. However, in WT and *βarr2*^*–/–*^, they were still shorter than the responses to the first stimulation retinas. By contrast, in *βarr1*^*–/–*^ responses to the second stimulation were similar to the first stimulation responses and at the same level compared to WT. Finally, we tested whether supplementation of *11-cis*-retinal *in vivo* would restore, at least in part, sustained melanopsin response in β*arr1*^*–/–*^ animals. After injection of *11-cis*-retinal subcutaneous (SQ), *βarr1*^*–/–*^ pups, previously shown to be insensitive to light (Figures 3D and 3F), displayed suppression of locomotor activity similar to that observed in WT pups (Figure 6D). The injection of *11-cis*-retinal did not further increase the photophobic reflex in WT pups.

## DISCUSSION

Here, we showed that β-arrestins are necessary for the unique property of ipRGCs to signal continuously under prolonged illumination. Both β-arrestins determine the melanopsin response time course. We demonstrated that the phosphorylation of a cluster of recently discovered Ser/Thr sites forms a binding site for both b-arrestin 1 and β-arrestin 2 and that this binding determines melanopsin response properties. Despite structure similarities and potential redundancies, β-arrestin 1 and β-arrestin 2 act preferentially at different steps of melanopsin photoresponse; β-arrestin 2 is necessary for melanopsin signal termination, whereas β-arrestin 1 is necessary for regeneration of the photopigment. However, partial redundancy between these 2 β-arrestins likely explains improved regeneration when *Opn4-F2A-βArr2* was expressed in *rd;Opn4*^*–/–*^ retinas (Figures 5D, S5E, and S5F).

Each mouse retina contains <5,000 melanopsin-expressing RGCs (mRGCs) in the inner retina, which is <0.01% of all rod and cone photoreceptors in the outer retina. This diminishingly small number of ipRGCs makes it difficult to use biochemical and stoichiometric techniques that are widely used to assess rhodopsin-arrestin (or any other assessor protein) interactions. Therefore, we used a set of complementary set of *in vitro*, *in vivo*, and behavioral assays to probe melanopsin-arrestin interaction in melanopsin photoresponses. A potential drawback of such an approach is that the stoichiometry of interacting molecules in a heterologous expression system may not be comparable to that in the native ipRGCs. To address this potential confusion, we took a complementary approach in which a hypothesis generated in cell line experiments is tested in the retina or in behavioral assays and vice versa (Figure S7A). Such cross-validation across experimental platforms minimizes the misinterpretation of experimental results.

Recording melanopsin photoresponses in retinas expressing melanopsin specifically in ipRGCs or in random RGCs, in individual β-arrestin-deficient mice, and in retinas in which melanopsin was co-expressed with β-arrestin offered a distinct experimental setup to test the roles of β-arrestin in ipRGC function. We observed prolonged responses (i.e., a high number of spikes and little desensitization) in *βarr2*^*–/–*^ mice. This higher magnitude and prolonged response after cessation of the light pulse is reminiscent of the responses from phosphorylation-deficient *Opn4*^*9A*^ mutant melanopsin (Mure et al., 2016). Further-more, both phosphorylation and arrestin binding improved the precision of deactivation. Accordingly, as seen in retinas expressing *Opn4*^*9A*^, *βarr2*^*–/–*^ retinas also showed excessive noise in response duration after cessation of the light pulse (Figure S7B). This wider spread in response duration (or noise in deactivation) was more pronounced in *βarr2*^*–/–*^ retinas than it was in β*arr1*^*–/–*^ retinas (Figure S7C). Since phosphorylation is a prerequisite for strong melanopsin-arrestin interaction for response deactivation (Figure 2B), similarities between photo-responses in *Opn4*^*9A*^ and *βarr2*^*–/–*^ retinas is best interpreted as a reduced efficiency in signal termination in *βarr2*^*–/–*^ retinas. Furthermore, in cell line assays, the co-expression of *βArr2* or *βArr2*^*R170E*^ (constitutively active mutant) reduced the melanop-sin signal more effectively than *βArr1* or *βArr1*^*R169E*^ (Figures 2C–2F). Thus, *βarr2* is likely the dominant arrestin for melanopsin photoresponse termination.

One of the signatures of melanopsin is the persistence of its response long after the stimulation has been extinguished. This has been called persistence in mRGCs (Do and Yau, 2010) and is reflected at the behavioral level by the post-illumination pupil response (PIPR) (Gamlin et al., 2007; Mure et al., 2009). We have shown previously that the structure of melanopsin, particularly its long C-terminal tail, contributes to slowing down its deactivation (Mure et al., 2016). Here, we showed that another contributor to this persistent response is β-arrestin 2. The bistable photopigments are characterized by the thermal stability of the active photoproduct and, consequently, the persistence of signaling after extinction of the stimulus. Melanopsin is a bistable or tristable photopigment (Mure et al., 2007; Emanuel and Do, 2015), making melanopsin functionally closer to insect rhodopsin than to mammalian rod or cone opsins. In fruit flies, bright blue light creates more metarhodopsin molecules than can be blocked by the available arrestin, leaving a surplus of active metarhodopsins that can maintain the phototransduction process (prolonged depolarizing afterpotential), even in complete darkness (Byk et al., 1993; Dolph et al., 1993). This suggests that low levels of β-arrestin 2 relative to melanopsin in ipRGCs may contribute to persistent photoresponse.

β-arrestin 1 deficiency, even if it does not seem to affect signal termination, results in a progressive loss of responsiveness to repeated 1-min light stimuli or long-duration illumination. Over-expression of β-arrestin 1 in random RGCs promotes regenera-tion, as is seen in retinas with Opn4-F2A-βArr1 (Figures 5D, S6E, and S6F). These results implicate b-arrestin 1 in melanopsin regeneration, in other words, the reconstitution of active photo-pigment (bound to *11-cis*-retinal), either via the visual retinoid cycle or by photoisomerization. Providing exogenous *11-cis*-retinal to *βarr1*^*–/–*^ retinas consistently restored sustained responses to a significant fraction of ipRGCs, as well as behavioral responses. While it is possible that part of the responsiveness restoration is due to the association of 11-*cis*-retinal with melanopsin that is synthetized *de novo,* protein turnover cannot completely explain this result, as very limited restoration is observed in WT and βarr2^*–/–*^ retinas. The variability of the effect of adding *11-cis*-retinal may reflect limited diffusion of the chromophore in this *ex vivo* preparation. This similarity of phenotype, rapid adaptation between *βarr1*^*–/–*^ retinas and retinas in which Opn4 is re-expressed in random RGCs, and its rescue by overexpressing *βarr1* suggests that the abundance of β-arrestin 1 or its relative expression compared to β-arrestin 2 differs between RGCs (lower) and ipRGC (higher).

Regeneration seems also to depend indirectly on β-arrestin 2, either because b-arrestin 1 must compete with β-arrestin 2 for binding and/or because previous melanopsin deactivation (including β-arrestin 2-dependent deactivation) is required to proceed to the next step of regeneration (as shown for other GPCRs) (Zhang et al., 1997). This was confirmed by the long 20-min stimulations, in which *βarr2*^*–/–*^ mutants initially (during the first minutes) displayed strong responses (similar to upon 1-min stimulation and consistently with their photophobic response), but they ultimately stopped responding. Modalities of interactions between the 2 β-arrestins remain to be clarified. GPCRs have been sorted into 2 main families, class A and class B, partly based on their interaction with arrestins. Class A GPCRs, including the β2AR (adrenergic receptor), have different affinities for different β-arrestin isoforms, resulting in sequential binding in different time windows (Oakley et al., 2000). Another possibility, since many GPCRs may function as dimers, is that functionally active dimeric melanopsin (Cameron and Robinson, 2014) may have 2 binding sites for arrestin recruitment.

**Figure 7.**
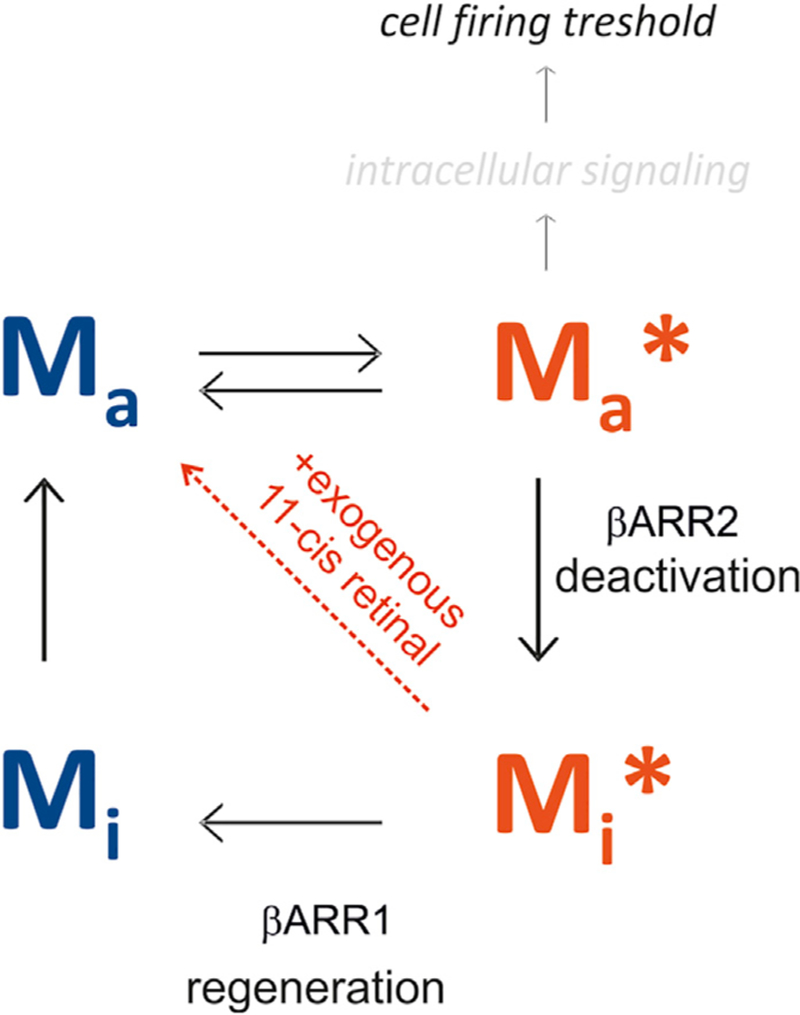
Both β-Arrestins Contribute Specifically to the Melanopsin Photocycle Proposed melanopsin photocycle model. Active melanopsin (M_a_) is photo-isomerized into activated melanopsin (M_a_*), the signaling form, upon photon absorption. Phosphorylation of M_a_* primes its binding by βArr2 and subse-quent deactivation (M_i_*, inactive melanopsin). βArr1 would then participate in melanopsin chromophore exchange and subsequent ipRGCs resensitization. Absorption of a photon from another wavelength may photoregenerate directly the Ma* into the Ma form. Exogenous 11-*cis*-retinal supply rescues the desensitization observed in β*arr1*-deficient mice.

In summary, the results presented in this article support the model depicted in Figure 7. Melanopsin photopigment upon photoactivation is phosphorylated and then bound by 2 different arrestins. β-arrestin 2 promotes the desensitization of active melanopsin, whereas β-arrestin 1 supports receptor regeneration. The ratios between melanopsin and the 2 β-arrestins determine the sustained response under tonic stimulation. Ectopic expression of melanopsin in random RGCs revealed that the relative expression of melanopsin and β-arrestins in native ipRGCs may contribute to their characteristic feature of reporting continuous illumination.

## STAR★METHODS

### CONTACT FOR REAGENT AND RESOURCE SHARING

Further information and requests for resources and reagents should be directed to and will be fulfilled by the Lead Contact, Satchin Panda (panda@salk.edu).

### EXPERIMENTAL MODEL AND SUBJECT DETAILS

#### Ethics Statement

All experiments were performed in accordance with the Institutional Animal Care and Use Committee (IACUC) guidelines of the Salk institute in compliance with the Animal Welfare Act and other federal and state statutes and regulations relating to animal experiments.

#### Mice

All animal care and procedures were approved by the Institutional Animal Care and Use Committed of the Salk Institute for Biological Studies. Mice were housed under 12 h light: 12 h dark (LD) cycles. Food and water were available *ad libitum*. Generation of *Opn4*^*–/–*^, *rd;Opn4*^*–/–*^ and *Opn4*^*cre/cre*^ mice were described previously (Hatori et al., 2008; Panda et al., 2002, 2003). C3H/HeJ strain (*rd*) carrying *Pdeb*^*rd1*^ mutation were obtained from the Jackson Laboratory. *Opn4*^*Cre*^ were bred with *rd* to generate *rd ;Opn4*^*cre/cre*^.

### METHOD DETAILS

#### PathHunter assay

β-arrestin is fused to a deletion mutant of β-galactosidase that is catalytically inactive, and GPCR is tagged with a small fragment derived from the deleted sequence of the enzyme (ProLink). Upon GPCR-β-arrestin interaction, the two parts of β-galactosidase are brought into close proximity, which results in cleavage of the substrate and generation of a chemiluminescent signal.

#### Mutant melanopsin clones

Mouse wild-type or mutant melanopsin were cloned into pAAV-EF1a-double floxed-hChR2(H134R)-mCherry-WPRE-HGHpA (Gradinaru et al., 2010) by using *AscI* and *NheI* restriction enzyme sites. The constructs were packaged into AAV2.2 serotype virus at the Salk Institute virus core following standard protocol. Viruses with high titer were used for intravitreal injections following published method (Lin et al., 2008).

#### Ca^2+^ release assays

CHO cells stably expressing human melanopsin (CHO^Opn4^) were treated with trypsin and seeded onto poly D-lysine coated Costar 384-well plates (12,000 cells/well) and incubated overnight in serum-free medium. For most experiments, 2 h prior to assay, the cells were exposed to 1,000 lux light from a white fluorescent light source at room temperature for 1 h. Cell medium was removed and cells were washed once with 70 mL of assay buffer (Hank’s Balanced Salt solution supplemented with 20 mM HEPES, 2.5 mM probenecid and 0.05% BSA). Cells were loaded with calcium indicator Fluo-4 AM (Molecular Probes) using a MultiDrop fluid dispenser and incubated for 1 h at 37 C in a CO_2_ incubator, and then washed three times with assay buffer. Light-induced increase in fluorescence was measured as described earlier (Jones et al., 2013; Pulivarthy et al., 2007).

#### AAV2/2 virus production and intraocular injection

These constructs are packaged into AAV2 serotype virus at the Salk Institute virus core. The packaged viruses were concentrated and purified in PBS and intravitreally administered as described in (Lin et al., 2008).

#### Western Blotting

Western blotting was perfomed as previously described (Benegiamo et al., 2018). For total protein extraction from the retina, 2 frozen retinas from the same mouse were mechanically homogenized in RIPA lysis buffer (10mM Tris-HCl pH8.0, 1mM EDTA, 1% Triton X-100, 0.1% SDS, 140mM NaCl and protease inhibitor cocktail). Samples were incubated at 4 C for 30 minutes with agitation and centrifuged at 13000rpm for 10 minutes at 4 C. The protein concentration in the supernatant was determined using the BCA assay (Pierce). After addition of Bolt Sample Reducing Agent (ThermoFisher), equal amounts of protein (40 mg) were heat-denatu-rated in Bolt LDS Sample Buffer, resolved by SDS-PAGE using Bolt 4%–12% Bis-Tris Plus Gels (ThermoFisher), and transferred to a nitrocellulose membrane using the iBlot Dry Blotting system (ThermoFisher). The membranes were blocked in 1XPBS 1% Casein Blocker (BioRad) diluted 1:10 for 1h at room temperature and then incubated with rabbit anti-OPN4 antiserum (against a peptide consisting of the 15 N-terminal amino acids of mouse melanopsin (Pulivarthy et al., 2007)) 1:200, b-arrestin 1/2 (mouse monoclonal, sc-74591) 1:100, and TBP (rabbit polyclonal, sc-273) 1:200. Alexa Fluor 680 conjugate anti-Rabbit IgG (ThermoFisher, A-10043) or alexa Fluor 680 conjugate anti-mouse IgG (Thermo Fisher, A21058) were used as secondary antibodies. Membrane-bound immune complexes were detected by Odyssey Imaging Systems (LI-COR Biosciences). Quantification was performed using Image Studio software (LI-COR Biosciences). Data were normalized to TBP protein expression.

#### Multi Electrodes Array (MEA) recording

After removal from the eye, a patch of retina about 4–10 mm^2^ will be mounted on a Multi-electrode array (Multichannel Systems, Reut-lingen, Germany), ganglion cell side down, and perfused with oxygenated Ames’ medium at 35 C supplemented with 20 mM CNQX and 50 mM D-APV to block glutamatergic transmission. The activity of ganglion cells is recorded via 256 electrodes 30 mm in diameter spaced every 100 mm apart and arranged in a 16 3 16 square grid (Multi Channels Systems MCS GmbH). Full-field visual stimuli at a flux of 5.10^12^ photons/cm^2^/s at the retina were presented during recordings using a high brightness LED (LuxeonStar 5, luxeonstar. com) with a peak wavelength of 480 nm. The current through the LED is controlled using custom electronics and software written in MATLAB (Mathworks, Natick, MA) and aligned with the physiological recording with a resolution of ± 100 ms. The signal is acquired from all 256 channels @ 10 kHz. Negative thresholds for spike detection are set at 5 times the standard deviation of the noise on each channel. Spike cutouts, consisting of 1 msec preceding and 2 msec after a supra threshold event, along with a time stamp of the trigger is written to hard disk. For each electrode, these spike cutouts are sorted into trains of a single cell after recording using Offline Sorter (Plexon, Denton, TX). Data analysis and display are performed using Neuroexplorer (Plexon) and custom software written in MATLAB.

11-cis-retinaldehyde was obtained through NEI (https://www.nei.nih.gov/funding/11_cis_retinal). For the chromophore rescue experiment, the regular recording medium was switched to the same Ames’ medium supplemented with 25 mM 11-cis-retinal dissolved in 0.1% ethanol.

#### Analysis

Generation of the LRAM: Light response adaptation map (LRAM) are generated to represent the alteration of the responses duration to repetition of identical stimuli (Figures 1 and 5). Our protocol comprises 4 different stimuli (0.1, 1, 10 and 60 s of 480 nm light delivered at 5.10^12^ph/cm^2^/s), each of which is repeated 5 time. For each genotype/model, we calculated the linear regression among repetitions of the same stimulus and use the surface function of MATLAB to interpolate between stimuli.

Adaptation rate: The adaptation rate (Figures 5D and 7B): we defined it as the slope of the linear regression of the parameters (dura-tion or number of spikes) in response to the repetition of identical 1min stimulations. It represents the relative variation of a response parameter from one stimulation to the other. It is calculated for each individual cells and expressed as average ± sem.

#### Negative phototaxis assay

WT, *βarr1*^*–/–*^ and *βarr2*^*–/–*^ pups aged from P7 to P9 were tested in a phototaxis assay (once) with the procedure modified from (Johnson et al., 2010). After the mouse pups were dark adapted for 1 h, a small (3 mm diameter) reflective dome sticker was placed on their head for video tracking and the pup was placed inside a transparent cylindrical plexiglass tube for 10 min. After the first 2 min under total darkness, the tube was illuminated for 6 min with succesive steps of bright mono-chromatic 480 nm light (2min each, 5.10^13^, 5.10^14^ and 5.10^15^ph/cm^2^/s). The tube was illuminated with 2 infrared (IR) LED bars (Environmental Light™) and the pup’s activity was video recorded for 10 min (2 min in dark, 6 min light ON and 2min after light OFF) with a Sony video camera equipped with an IR filter. Digital movies were then analyzed offline with a custom centroid detection-based program implemented in MATLAB (Mathworks). We extracted the distance covered by the head in 1 s bins that allowed us to obtain the pups activity profile during the recording as well as to compare the mean activity before and after light on. Pups that didn’t show significant activity (5 pixels/bin threshold) during the 2 min before light ON were excluded from the analysis.

For the *11-cis* retinal supplementation assay, the pups were injected subcutaneously between the shoulder blades with either 11-*cis* retinal (approximately 25 mg/kg mouse in 150 μL vehicle [10% ethanol and 0.9% NaCl]) or the vehicule only 10min before the start of the recording while in darkness. Each pup’s activity was video recorded for 4 min; 2 min in dark followed by 2 min under monochromatic 480 nm light (5.10^14^ ph/cm^2^/s). The distance covered by the head of the animal during the light period was normal-ized to the distance covered in the dark period and then averaged across animals.

#### Pupillary light reflex (PLR)

Mice were implanted with an acrylic headpost. After at least 1 week of recovery from the headposting surgery, they were tested for PLR. Before the recordings, mice were briefly anesthetized with isofluorane and restrained in a custom-made animal holder. The animal holder was placed inside a light-tight box with the left eye apposed against an opening of an integrating sphere. Light from a 300 Watt Xenon Arc lamp light source (Sutter Instrument, Novato, CA, USA) was filtered, collimated and delivered to the integrating sphere through a liquid light guide. An inline 480 nm filter, a filter wheel with a neutral density filter and a Lambda 10–3 optical filter changer with SmartShutter™ were used to control the spectral quality, intensity, and duration of light. Light intensity was measured with a Melles Griot power meter. The mouse’s right eye was illuminated by an IR LED and recorded with a high precision LINX video camera (Imperx Inc.) equipped with an IR filter at a sample rate of 30Hz. We recorded 5 min sequences consisting of 1 min of darkness, 1 min of monochromatic 480 nm light (5.5 3 10^12^ ph/cm^2^/s) and finally 3 min of darkness. Stimulations and recordings are synchronized with a custom Labview (National Instruments) program. Digital movies of pupil constriction were then analyzed offline with a custom MATLAB program. We extracted the pupil diameter. The mean diameter measured during the first period of darkness (1 min) of each sequence served as the baseline for normalization of the recordings.

### QUANTIFICATION AND STATISTICAL ANALYSIS

All data are expressed as means ± SEM unless stated otherwise. Statistical tests used are stated in the figures legends. Differences between groups were considered statistically significant for p < 0.05.

## Supplementary Material

1

## Figures and Tables

**Table:** KEY RESOURCES TABLE

REAGENT or RESOURCE	SOURCE	IDENTIFIER
Antibodies		

Rabbit anti-OPN4 antiserum	Salk Institute, Pulivarthy et al.,2007	RRID: AB_2571553
Mouse monoclonal anti b-arrestin 1/2	Santa Cruz	sc-74591,
Normal rabbit anti-IgG	Santa Cruz	sc-2027, RRID: AB_737197
Rabbit polyclonal anti-TBP	Santa Cruz	sc-273
Alexa Fluor 680 conjugate anti-Rabbit IgG	Thermo Fisher	A-10043
Alexa Fluor 680 conjugate anti-mouse IgG	Thermo Fisher	A-21058

Bacterial and Virus Strains		

pAAV2/2-MSC8-mOpn4 WT-1d4tag	Salk Institute virus core, Mure et al., 2016	N/A
pAAV2/2-MSC8-mOpn4 397truncation-1d4tag	Salk Institute virus core, Mure et al., 2016	N/A
pAAV2/2-MSC8-mOpn4 9Amutation-1d4tag	Salk Institute virus core, Mure et al., 2016	N/A
AAV2-hSyn-mOpn4-F2A-mβArr1a-flag	Salk Institute virus core, this study	N/A
AAV2-hSyn-mOpn4-F2A-mβArr2-flag	Salk Institute virus core, this study	N/A

Chemicals, Peptides, and Recombinant Proteins		

*11 cis* retinal	National Eye Institute	https://nei.nih.gov/funding/11_cis_retinal
Fluo-4 AM	Molecular Probes	F14201

Critical Commercial Assays		

PathHunter® β-Arrestin Assays	DiscoverX	https://www.discoverx.com/arrestin

Experimental Models: Cell Lines		

CHO cells stably expressing human melanopsin (CHO^Opn4^)	Jones et al., 2013	N/A
Experimental Models: Organisms/Strains		
Arrb1 KO	The Jackson Laboratory (Robert Lefkowitz)	N/A
Arrb1 KO	The Jackson Laboratory (Robert Lefkowitz)	N/A
rd (C3H/HeJ)	The Jackson Laboratory	N/A
rd;b1	This study	N/A
rd;b2	This study	N/A
Opn4cre/cre	This study	RRID: MGI_3798479
rd;Opn4cre/cre	This study	RRID:MGI_5754375

Software and Algorithms		

Prism	GraphPad	RRID: SCR_015807, https://www.graphpad.com
MATLAB	MathWorks	RRID: SCR_001622, https://www.mathworks.com/products/matlab.html
Plexon Offline Sorter	Plexon	RRID:SCR_000012, https://plexon.com/products/offline-sorter/
NeuroExplorer	Plexon	RRID:SCR_001818, http://plexon.com/products/neuroexplorer
MC Rack	Multichannelsystems	RRID:SCR_014955, https://www.multichannelsystems.com/software/mc-rack
LI-COR Image Studio	LI-COR Biosciences	RRID: SCR_015795, https://www.licor.com/bio/products/software/image_studio/

## Highlights

- Phosphorylation of melanopsin upon activation primes binding to β-arrestin 1 and 2
- Each β-arrestin regulates different aspects of melanopsin photoresponses
- β-Arrestin 2 mediates signal termination, and β-arrestin 1 mediates melanopsin recycling
- Both β-arrestins are necessary to sustain melanopsin continuous response

## References

[R1] Benegiamo G, Mure LS, Erikson G, Le HD, Moriggi E, Brown SA, and Panda S (2018). The RNA-binding protein NONO coordinates hepatic adap-tation to feeding. Cell Metab, 27, e7. 10.1016/j.cmet.2017.12.010 PMC6996513 29358041

[R2] Blasic JR Jr., Matos-Cruz V, Ujla D, Cameron EG, Hattar S, Halpern ME, and Robinson PR (2014). Identification of critical phosphorylation sites on the carboxy tail of melanopsin. Biochemistry 53, 2644–2649. 24678795 10.1021/bi401724r PMC4010260

[R3] Bohn LM, Lefkowitz RJ, Gainetdinov RR, Peppel K, Caron MG, and Lin FT (1999). Enhanced morphine analgesia in mice lacking beta-arrestin 2. Science 286, 2495–2498. 10617462 10.1126/science.286.5449.2495

[R4] Brown TM, Gias C, Hatori M, Keding SR, Semo M, Coffey PJ, Gigg J, Piggins HD, Panda S, and Lucas RJ (2010). Melanopsin contributions to irradiance coding in the thalamo-cortical visual system. PLoS Biol 8, e1000558. 21151887 10.1371/journal.pbio.1000558 PMC2998442

[R5] Byk T, Bar-Yaacov M, Doza YN, Minke B, and Selinger Z (1993). Reg-ulatory arrestin cycle secures the fidelity and maintenance of the fly photore-ceptor cell. Proc. Natl. Acad. Sci. USA 90, 1907–1911. 8446607 10.1073/pnas.90.5.1907 PMC45989

[R6] Cameron EG, and Robinson PR (2014). β-Arrestin-dependent deactivation of mouse melanopsin. PLoS One 9, e113138. 25401926 10.1371/journal.pone.0113138 PMC4234672

[R7] Celver J, Vishnivetskiy SA, Chavkin C, and Gurevich VV (2002). Conservation of the phosphate-sensitive elements in the arrestin family of proteins. J. Biol. Chem 277, 9043–9048. 11782458 10.1074/jbc.M107400200

[R8] Conner DA, Mathier MA, Mortensen RM, Christe M, Vatner SF, Seid-man CE, and Seidman JG (1997). beta-Arrestin1 knockout mice appear normal but demonstrate altered cardiac responses to beta-adrenergic stimu-lation. Circ. Res 81, 1021–1026. 9400383 10.1161/01.res.81.6.1021

[R9] Do MTH, and Yau K-W (2010). Intrinsically photosensitive retinal ganglion cells. Physiol. Rev 90, 1547–1581. 20959623 10.1152/physrev.00013.2010 PMC4374737

[R10] Dolph PJ, Ranganathan R, Colley NJ, Hardy RW, Socolich M, and Zuker CS (1993). Arrestin function in inactivation of G protein-coupled recep-tor rhodopsin in vivo. Science 260, 1910–1916. 8316831 10.1126/science.8316831

[R11] Eglen RM (2002). Enzyme fragment complementation: a flexible high throughput screening assay technology. Assay Drug Dev. Technol 1, 97–104. 15090161 10.1089/154065802761001356

[R12] Emanuel AJ, and Do MTH (2015). Melanopsin tristability for sustained and broadband phototransduction. Neuron 85, 1043–1055. 25741728 10.1016/j.neuron.2015.02.011 PMC4351474

[R13] Emanuel AJ, Kapur K, and Do MTH (2017). Biophysical variation within the M1 type of ganglion cell photoreceptor. Cell Rep 21, 1048–1062. 29069587 10.1016/j.celrep.2017.09.095 PMC5675019

[R14] Fu Y, Zhong H, Wang M-HH, Luo D-G, Liao H-W, Maeda H, Hattar S, Frishman LJ, and Yau K-W (2005). Intrinsically photosensitive retinal ganglion cells detect light with a vitamin A-based photopigment, melanopsin. Proc. Natl. Acad. Sci. USA 102, 10339–10344. 16014418 10.1073/pnas.0501866102 PMC1177370

[R15] Gainetdinov RR, Premont RT, Bohn LM, Lefkowitz RJ, and Caron MG (2004). Desensitization of G protein-coupled receptors and neuronal functions. Annu. Rev. Neurosci 27, 107–144. 15217328 10.1146/annurev.neuro.27.070203.144206

[R16] Gamlin PDR, McDougal DH, Pokorny J, Smith VC, Yau K-W, and Dacey DM (2007). Human and macaque pupil responses driven by melanop-sin-containing retinal ganglion cells. Vision Res 47, 946–954. 17320141 10.1016/j.visres.2006.12.015 PMC1945238

[R17] Gradinaru V, Zhang F, Ramakrishnan C, Mattis J, Prakash R, Diester I, Goshen I, Thompson KR, and Deisseroth K (2010). Molecular and cellular approaches for diversifying and extending optogenetics. Cell 141, 154–165. 20303157 10.1016/j.cell.2010.02.037 PMC4160532

[R18] Hatori M, Le H, Vollmers C, Keding SR, Tanaka N, Buch T, Waisman A, Schmedt C, Jegla T, and Panda S (2008). Inducible ablation of mela-nopsin-expressing retinal ganglion cells reveals their central role in non-image forming visual responses. PLoS One 3, e2451. 18545654 10.1371/journal.pone.0002451 PMC2396502

[R19] Hattar S, Lucas RJ, Mrosovsky N, Thompson S, Douglas RH, Hankins MW, Lem J, Biel M, Hofmann F, Foster RG, and Yau KW (2003). Mel-anopsin and rod-cone photoreceptive systems account for all major acces-sory visual functions in mice. Nature 424, 76–81. 12808468 10.1038/nature01761 PMC2885907

[R20] Hayter EA, and Brown TM (2018). Additive contributions of melanopsin and both cone types provide broadband sensitivity to mouse pupil control. BMC Biol 16, 83. 30064443 10.1186/s12915-018-0552-1 PMC6066930

[R21] Isoldi MC, Rollag MD, Castrucci A.M. de L., and Provencio I. (2005). Rhabdomeric phototransduction initiated by the vertebrate photopigment melanopsin. Proc. Natl. Acad. Sci. USA 102, 1217–1221. 15653769 10.1073/pnas.0409252102 PMC545850

[R22] Johnson J, Wu V, Donovan M, Majumdar S, Renterı a, R.C., Porco T, Van Gelder RN, and Copenhagen DR. (2010). Melanopsin-dependent light avoidance in neonatal mice. Proc. Natl. Acad. Sci. USA 107, 17374–17378. 20855606 10.1073/pnas.1008533107 PMC2951438

[R23] Jones KA, Hatori M, Mure LS, Bramley JR, Artymyshyn R, Hong S-P, Marzabadi M, Zhong H, Sprouse J, Zhu Q, (2013). Small-molecule antagonists of melanopsin-mediated phototransduction. Nat. Chem. Biol 9, 630–635. 23974117 10.1038/nchembio.1333 PMC3839535

[R24] Keenan WT, Rupp AC, Ross RA, Somasundaram P, Hiriyanna S, Wu Z, Badea TC, Robinson PR, Lowell BB, and Hattar SS (2016). A visual circuit uses complementary mechanisms to support transient and sustained pupil constriction. eLife 5, e15392. 27669145 10.7554/eLife.15392 PMC5079752

[R25] Kovoor A, Celver J, Abdryashitov RI, Chavkin C, and Gurevich VV (1999). Targeted construction of phosphorylation-independent beta-arrestin mutants with constitutive activity in cells. J. Biol. Chem 274, 6831–6834. 10066734 10.1074/jbc.274.11.6831

[R26] LeGates TA, Fernandez DC, and Hattar S (2014). Light as a central modulator of circadian rhythms, sleep and affect. Nat. Rev. Neurosci 15, 443–454. 24917305 10.1038/nrn3743 PMC4254760

[R27] Lin B, Koizumi A, Tanaka N, Panda S, and Masland RH (2008). Restoration of visual function in retinal degeneration mice by ectopic expression of melanopsin. Proc. Natl. Acad. Sci. USA 105, 16009–16014. 18836071 10.1073/pnas.0806114105 PMC2572922

[R28] Lucas RJ, Hattar S, Takao M, Berson DM, Foster RG, and Yau K-W (2003). Diminished pupillary light reflex at high irradiances in melanopsin-knockout mice. Science 299, 245–247. 12522249 10.1126/science.1077293

[R29] Milner ES, and Do MTH (2017). A population representation of absolute light intensity in the mammalian retina. Cell 171, 865–876.e16. 28965762 10.1016/j.cell.2017.09.005 PMC6647834

[R30] Moore CAC, Milano SK, and Benovic JL (2007). Regulation of receptor trafficking by GRKs and arrestins. Annu. Rev. Physiol 69, 451–482. 17037978 10.1146/annurev.physiol.69.022405.154712

[R31] Mrosovsky N, and Hattar S (2003). Impaired masking responses to light in melanopsin-knockout mice. Chronobiol. Int 20, 989–999. 14680139 10.1081/cbi-120026043

[R32] Mure LS, Rieux C, Hattar S, and Cooper HM (2007). Melanopsin-dependent nonvisual responses: evidence for photopigment bistability in vivo. J. Biol. Rhythms 22, 411–424. 17876062 10.1177/0748730407306043 PMC2789279

[R33] Mure LS, Cornut P-L, Rieux C, Drouyer E, Denis P, Gronfier C, and Cooper HM (2009). Melanopsin bistability: a fly’s eye technology in the human retina. PLoS One 4, e5991. 19551136 10.1371/journal.pone.0005991 PMC2695781

[R34] Mure LS, Hatori M, Zhu Q, Demas J, Kim IM, Nayak SK, and Panda S (2016). Melanopsin-encoded response properties of intrinsically photosensitive retinal ganglion cells. Neuron 90, 1016–1027. 27181062 10.1016/j.neuron.2016.04.016 PMC4891235

[R35] Nayak SK, Jegla T, and Panda S (2007). Role of a novel photopigment, melanopsin, in behavioral adaptation to light. Cell. Mol. Life Sci 64, 144–154. 17160354 10.1007/s00018-006-5581-1 PMC11136037

[R36] Oakley RH, Laporte SA, Holt JA, Caron MG, and Barak LS (2000). Differential affinities of visual arrestin, beta arrestin1, and beta arrestin2 for G protein-coupled receptors delineate two major classes of receptors. J. Biol. Chem 275, 17201–17210. 10748214 10.1074/jbc.M910348199

[R37] Panda S, Sato TK, Castrucci AM, Rollag MD, DeGrip WJ, Hogenesch JB, Provencio I, and Kay SA (2002). Melanopsin (Opn4) requirement for normal light-induced circadian phase shifting. Science 298, 2213–2216. 12481141 10.1126/science.1076848

[R38] Panda S, Provencio I, Tu DC, Pires SS, Rollag MD, Castrucci AM, Pletcher MT, Sato TK, Wiltshire T, Andahazy M, (2003). Melanopsin is required for non-image-forming photic responses in blind mice. Science 301, 525–527. 12829787 10.1126/science.1086179

[R39] Panda S, Nayak SK, Campo B, Walker JR, Hogenesch JB, and Jegla T (2005). Illumination of the melanopsin signaling pathway. Science 307, 600–604. 15681390 10.1126/science.1105121

[R40] Pulivarthy SR, Tanaka N, Welsh DK, De Haro L, Verma IM, and Panda S (2007). Reciprocity between phase shifts and amplitude changes in the mammalian circadian clock. Proc. Natl. Acad. Sci. USA 104, 20356–20361. 18077393 10.1073/pnas.0708877104 PMC2154435

[R41] Qiu X, Kumbalasiri T, Carlson SM, Wong KY, Krishna V, Provencio I, and Berson DM (2005). Induction of photosensitivity by heterologous expression of melanopsin. Nature 433, 745–749. 15674243 10.1038/nature03345

[R42] Rao S, Chun C, Fan J, Kofron JM, Yang MB, Hegde RS, Ferrara N, Copenhagen DR, and Lang RA (2013). A direct and melanopsin-dependent fetal light response regulates mouse eye development. Nature 494, 243–246. 23334418 10.1038/nature11823 PMC3746810

[R43] Saari JC (2012). Vitamin A metabolism in rod and cone visual cycles. Annu. Rev. Nutr 32, 125–145. 22809103 10.1146/annurev-nutr-071811-150748

[R44] Sekaran S, Lupi D, Jones SL, Sheely CJ, Hattar S, Yau K-W, Lucas RJ, Foster RG, and Hankins MW (2005). Melanopsin-dependent photore-ception provides earliest light detection in the mammalian retina. Curr. Biol 15, 1099–1107. 15964274 10.1016/j.cub.2005.05.053 PMC4316668

[R45] Sexton TJ, and Van Gelder RN (2015). G-protein coupled receptor kinase 2 minimally regulates melanopsin activity in intrinsically photosensitive retinal ganglion cells. PLoS One 10, e0128690. 26069965 10.1371/journal.pone.0128690 PMC4467020

[R46] Somasundaram P, Wyrick GR, Fernandez DC, Ghahari A, Pinhal CM, Simmonds Richardson M., Rupp AC, Cui L, Wu Z, Brown RL, (2017). C-terminal phosphorylation regulates the kinetics of a subset of mela-nopsin-mediated behaviors in mice. Proc. Natl. Acad. Sci. USA 114, 2741–2746. 28223508 10.1073/pnas.1611893114 PMC5347544

[R47] Szymczak AL, Workman CJ, Wang Y, Vignali KM, Dilioglou S, Vanin EF, and Vignali DAA (2004). Correction of multigene deficiency in vivo us-ing a single ‘self-cleaving’ 2A peptide-based retroviral vector. Nat. Biotechnol 22, 589–594. 15064769 10.1038/nbt957

[R48] Tu DC, Zhang D, Demas J, Slutsky EB, Provencio I, Holy TE, and Van Gelder RN (2005). Physiologic diversity and development of intrinsically photosensitive retinal ganglion cells. Neuron 48, 987–999. 16364902 10.1016/j.neuron.2005.09.031

[R49] Zhang J, Barak LS, Winkler KE, Caron MG, and Ferguson SS (1997). A central role for beta-arrestins and clathrincoated vesicle-mediated endocytosis in beta2-adrenergic receptor resensitization. Differential regulation of receptor resensitization in two distinct cell types. J. Biol. Chem 272, 27005–27014. 9341139 10.1074/jbc.272.43.27005

[R50] Zhao X, Pack W, Khan NW, and Wong KY (2016). Prolonged inner retinal photoreception depends on the visual retinoid cycle. J. Neurosci 36, 4209–4217. 27076420 10.1523/JNEUROSCI.2629-14.2016 PMC4829646

