# Supplementary material for: Sustained Melanopsin Photoresponse Is Supported by Specific Roles of β-Arrestin 1 and 2 in Deactivation and Regeneration of Photopigment
Source: Cell Rep. Author manuscript; Available in PMC 2019 Mar 1. (PMC6396282; doi:10.1016/j.celrep.2018.11.008)
Supplement: 1 [file NIHMS1012693-supplement-1.pdf]

Cell Reports, Volume *25*

Supplemental Information

Sustained Melanopsin Photoresponse Is Supported by Specific Roles of β-Arrestin 1 and 2 in Deactivation and Regeneration of Photopigment

Ludovic S. Mure, Megumi Hatori, Kiersten Ruda, Giorgia Benegiamo, James Demas, and Satchidananda Panda

Supplementary Information

Figure S1

Figure S2

Figure S3

Figure S4

Figure S5

Figure S6

Figure S7

Figure S1

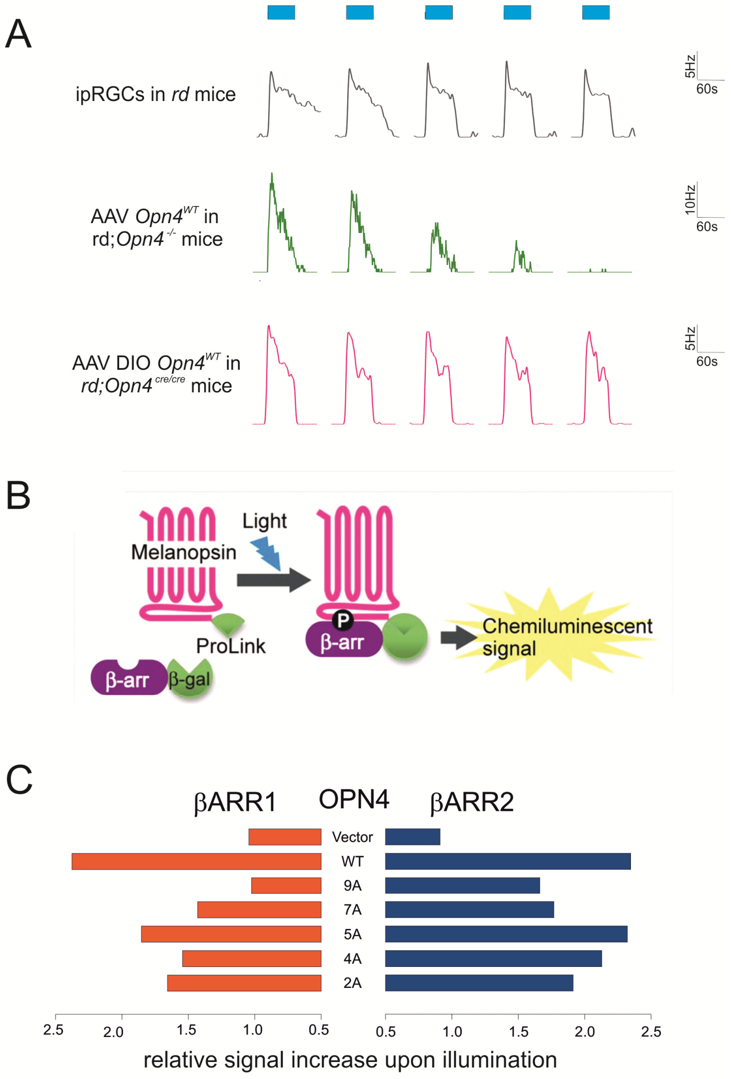

Figure S1 (related to Figure 1 and 2): (**A**) Representative example of individual ipRGCs from rd mice (upper panel, *grey*), RGCs from rd;Opn4$^{-/-}$mice transduced with Opn4^{WT} (middle panel, *green*), and ipRGCs from rd;Opn4$^{cre/cre}$ mice conditionally transduced with Opn4^{WT} (lower panel, *pink*) response to repetition of the same 60s light stimulus (blue bars, 480 nm, 5.10^{12} photons/cm2·s). **(B)** Partial coding sequence of β-galactosidase (β-gal) reporter is fused to mammalian βArr1or 2, and a complementary peptide (ProLink) is fused to the C-terminus of mOpn4. Upon photoactivation, chimeric β-arrestin is recruited to the activated melanopsin, the partial β-gal binds to the ProLink tag on Opn4 and the functional β-gal enzyme is reconstituted. The activity of the functional enzyme quantified by the chemiluminescent substrate is a measure of melanopsin-arrestin complex. **(C)** Light-dependent interaction between Opn4^{WT} and phosphodefient Opn4 mutants and β arrestins: ratio of the bioluminescent signal measured after light stimulation or darkness.

Figure S2

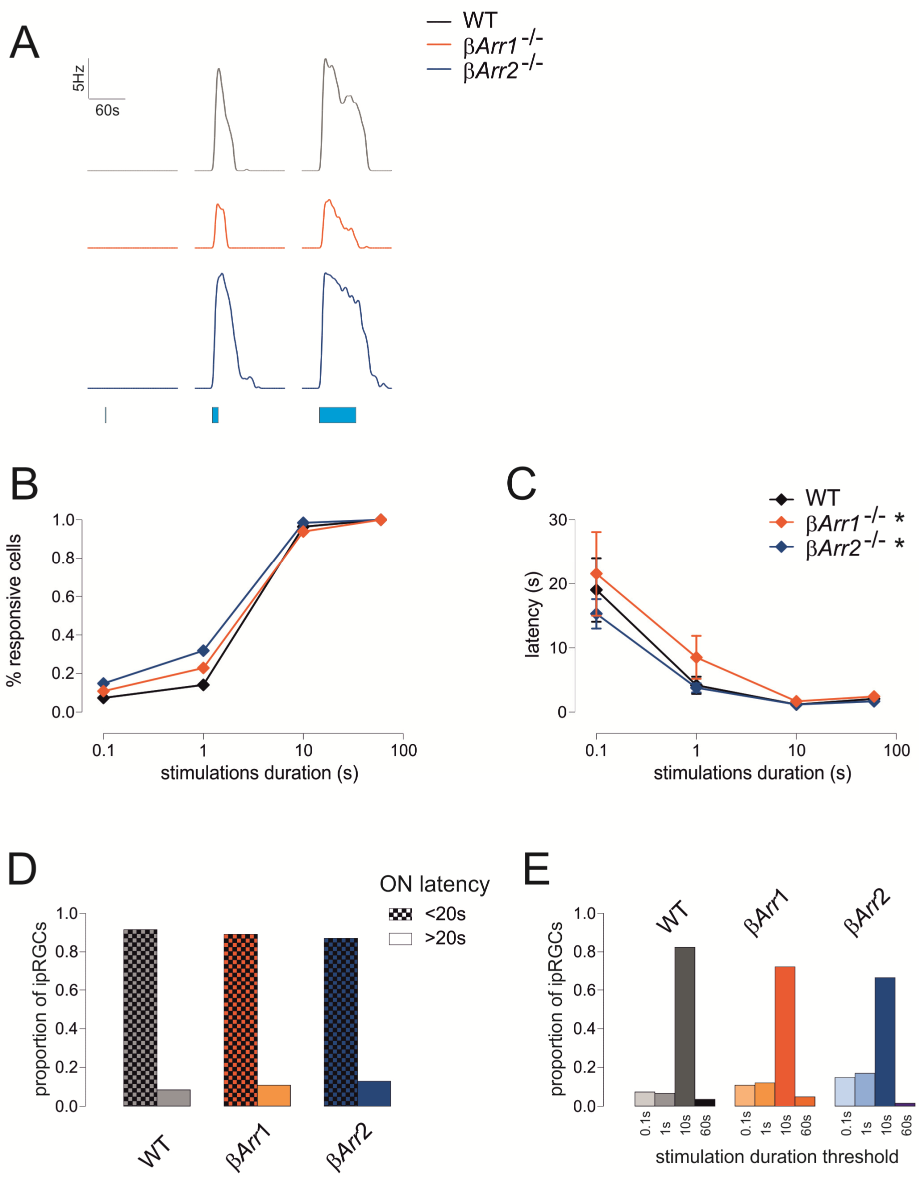

Figure S2 (related to Figure 3): Light responses from WT (n=284), *βArr1*$^{-/-}$ (n=83), and *βArr2*$^{-/-}$ (n=458) pups retinas (100ms, 1s, 10s and 1min, 488 nm, 5.10^{12} photons/cm^2·s): traces of individual cell responses to 10 and 60s of light (**A**), proportion of responsive cells (**B**) and response latency (**C**, ANOVA, p=0.016 and 0.023 for *βArr1*$^{-/-}$ and *βArr2*$^{-/-}$ respectively). (**D, E**) Neonatal ipRGCs subgrouped according to their latency to ON activation (**D**) and stimulation duration threshold (minimum stimulus length to obtain a response, **E**).

Figure S3

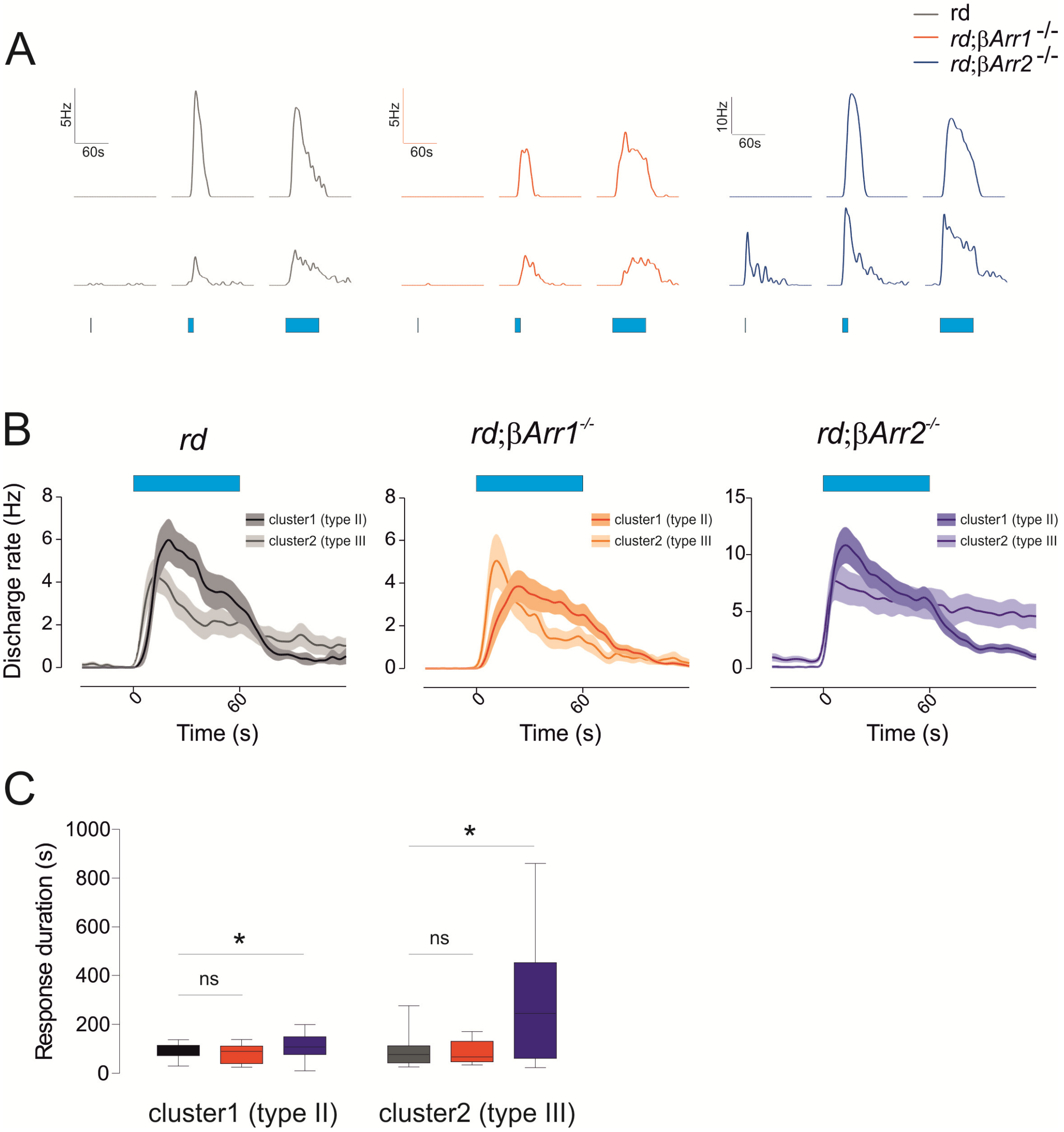

Figure S3 (related to Figure 4): Light responses from *rd* (n=32), *rd;βArr1$^{-/-}$* (n=32), and *rd;βArr2$^{-/-}$* (n=52) adult mice retinas (100ms, 1s, 10s and 1min, 488 nm, 5.10^{12} photons/cm^2·s): (**A**) traces of individual cell responses to 1, 10 and 60s of light (blue bars), (**B**) k-means clustering of *rd, rd;βArr1$^{-/-}$* and *rd;βArr2$^{-/-}$* mice responses to 1min light stimulation (488 nm, 5.10^{12} photons/cm^2·s, blue bar) and (**C**) median duration of the response for each cluster (whiskers 5-95 percentile, unpaired t-test).

Figure S4

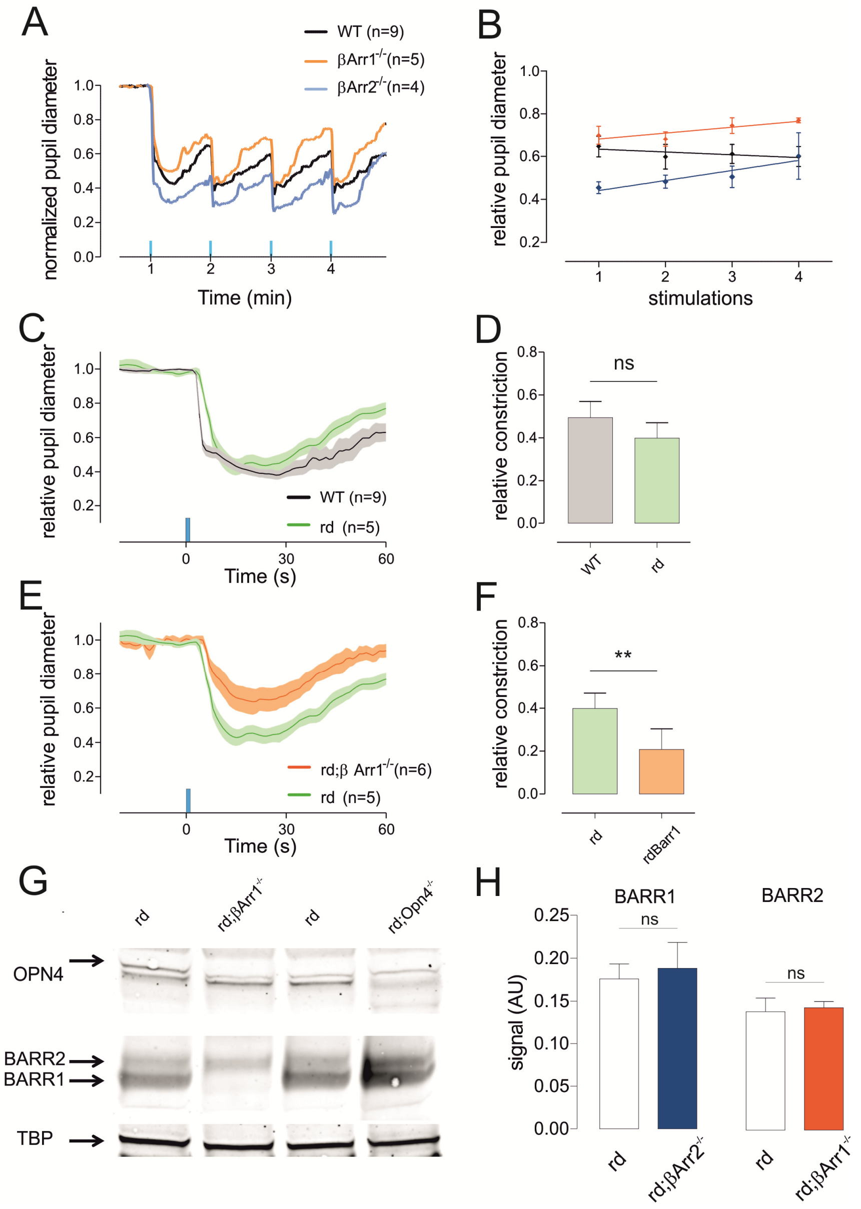

Figure S4 (related to Figure 4): (**A**) Average PLR traces in response to 4 successive 1s stimuli (1.10^{14} photons/cm^2/s, 488 nm) delivered 1min apart and (**B**) linear regression of the corresponding relaxation 1min after each 4 1s stimulations. Average PLR traces and constriction (for 1min following the stimulus) in response to a 1s stimulus (1.10^{14} photons/cm^2/s, 488 nm) in WT and *rd* mice (**C, D**) and in *rd* and *rd;βArr1$^{-/-}$* mice (**E, F**) (**p<0.01, one tailed Student t test). Protein expression by western blot analysis: (**G**) Example of blot and (**H**) average βARR 1 and 2 abundancy respectively in *rd;βArr2$^{-/-}$* and *rd;βArr1$^{-/-}$* mice (n=3).

Figure S5

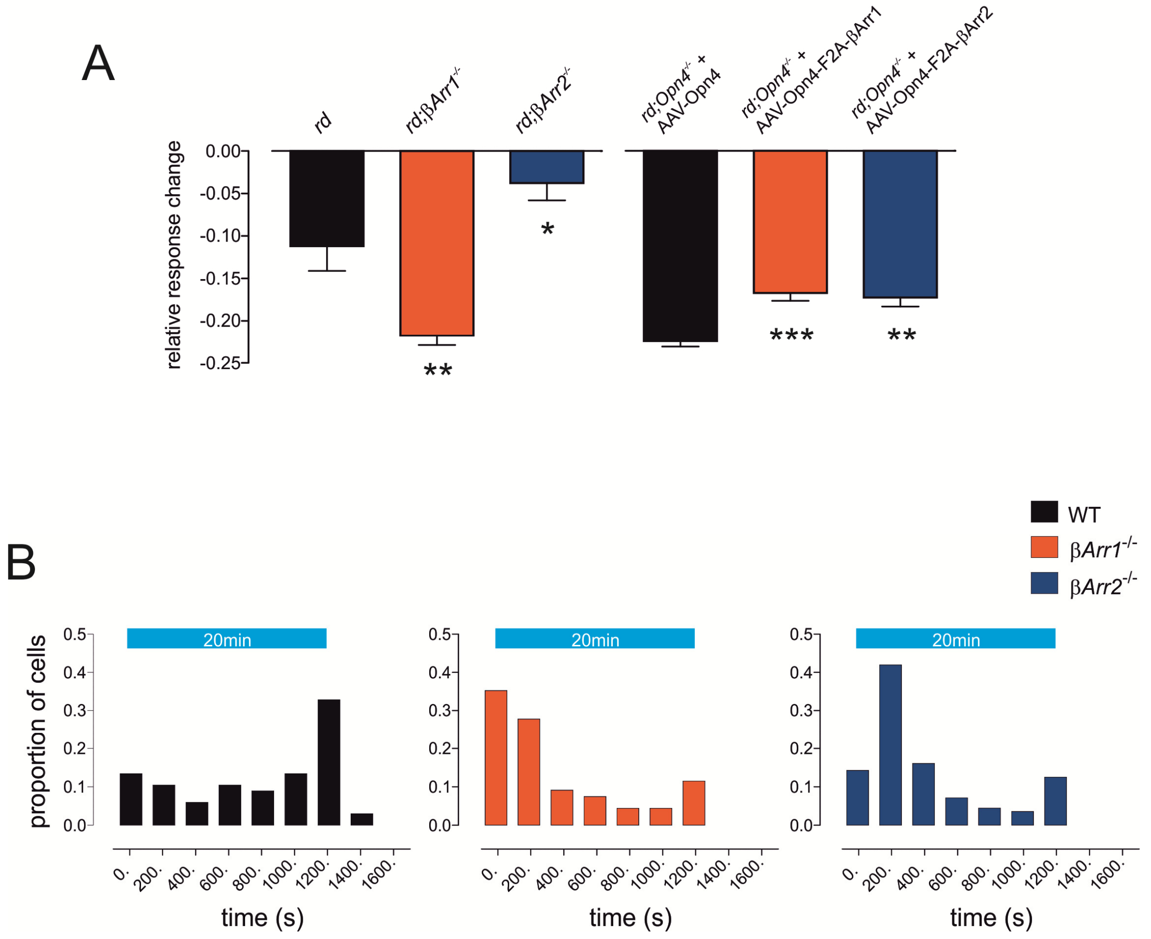

Figure S5 (related to Figure 5): (**A**) Adaptation rate of the number of spikes per response (average +/- SEM) observed in the beta arrestins deficient retina model (*rd* compared to *rd; βArr1$^{-/-}$* and *rd; βArr2$^{-/-}$*) and beta arrestin overexpression (AAV Opn4^{WT} compared to AAV Opn4^{WT}-F2A-βArr1 or Opn4^{WT}-F2A-βArr 2) observed in response to 5 repetitions of an identical 1 min light stimulus (MEA, 480 nm, 5.10^{12} photons/cm^{2}·s) (t-test, A2 vs A2-F2A-βArr2, p= 0.002 **; A2 vs A2-F2A-βArr1, p= 2.5E-06 ***; *rd* vs *rd; βArr1$^{-/-}$*, p= 0.002 **; *rd* vs *rd; βArr2$^{-/-}$*, p= 0.03 *). **(B)** Distribution of the duration of the responses from ipRGCs of WT, *βArr1$^{-/-}$*, and *βArr2$^{-/-}$* pups retinas stimulated continuously for 20 min (488 nm, 5.10^{12} photons/cm^{2}·s).

Figure S6

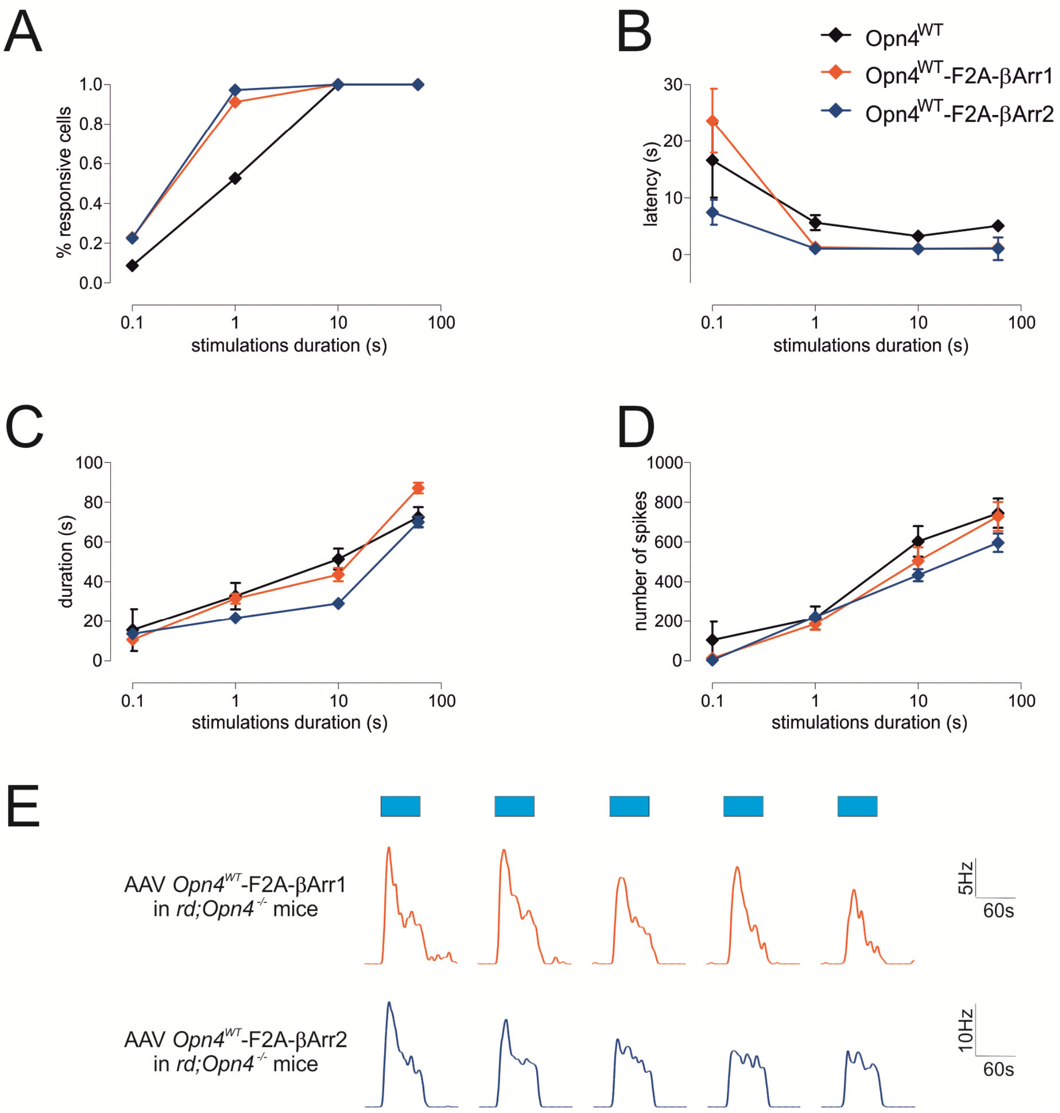

Figure S6 (related to Figure 6): Light responses from *rd*;*Opn4*$^{-/-}$ mice retinas transduced with Opn4^{WT}(n=57), Opn4^{WT}-*F2A-βArr1* (n=45), and Opn4^{WT} -*F2A-βArr2* (n=111) (100ms, 1s, 10s and 1min, 488 nm, 5.10^{12} photons/cm^{2}·s): proportion of responsive cells (**A**), response latency (**B,** Opn4^{WT}-*F2A-βArr2* ***) response duration (**C,** Opn4^{WT}-*F2A-βArr2* *), number of spikes (**D**) and representative example of individual RGCs from rd;Opn4$^{-/-}$mice transduced with Opn4^{WT}-F2A- *βArr1* or Opn4^{WT}-F2A- *βArr2* (**E**).

Figure S7

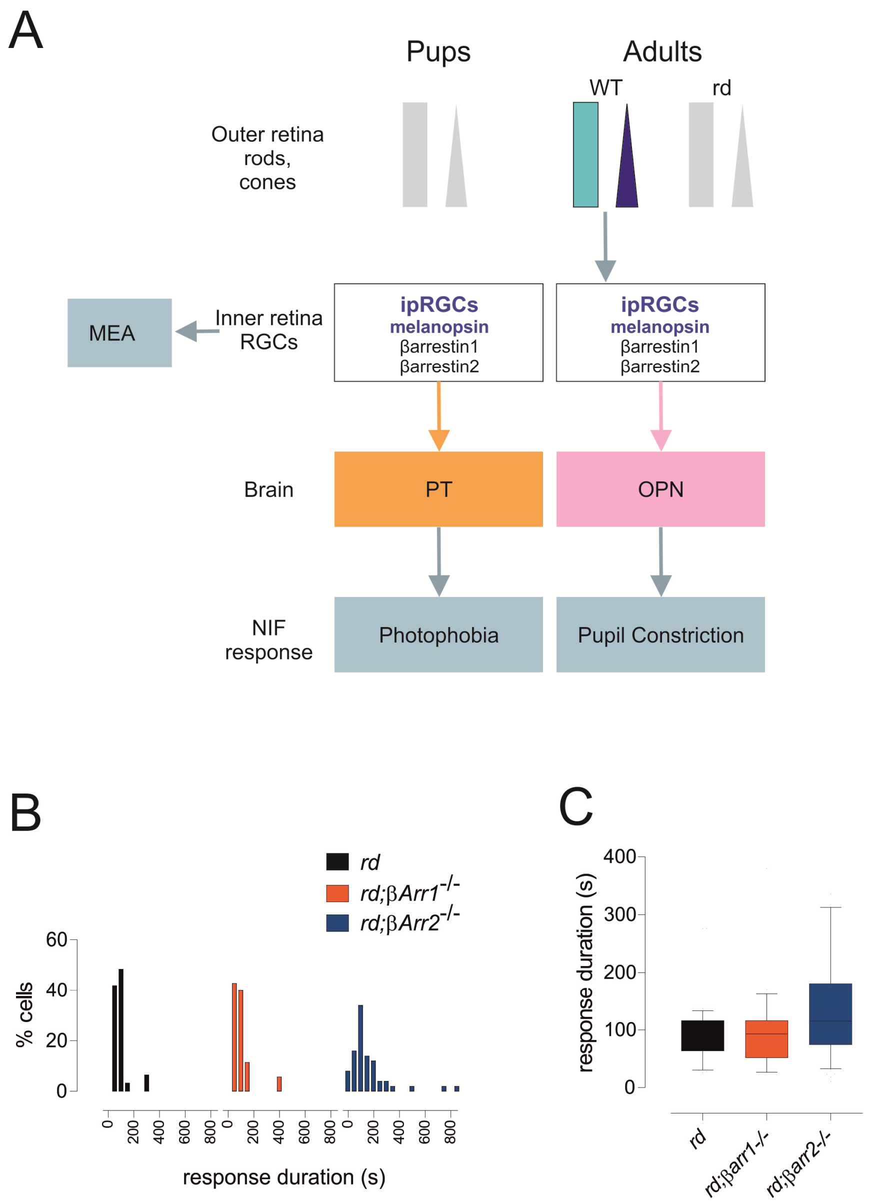

Figure S7 (related to Figure 7): (A) Circuits underlying the different non-visual light responses reported in this study. The ipRGCs integrates melanopsin and rods/cones (nonfunctional in the pups) signals and project to the Posterior thalamus (PT) and the Olivary pretectal nucleus (OPN), the brain centers mediating respectively photophobia and the PLR. (**B)** Distribution of the durations of the responses from ipRGCs of *rd*, *rd;βArr1* and *rd;βArr2* retinas to 1 min stimulation. **(C)** Dispersion of the durations of the responses from ipRGCs of *rd*, *rd;βArr1* and *rd;βArr2* retinas to 1 min stimulation (median, 10-90 percentiles wiskers).
